# Critical shortage of gastrointestinal physician density in the USA: impact on mortality from upper gastrointestinal bleeding

**DOI:** 10.3389/fmed.2026.1812471

**Published:** 2026-06-15

**Authors:** Abuoma Cherry Ekpendu, Muhammad Sohaib Asghar, Waqar Qureshi, Rohin Patel, Pankajkumar Patel, Chad K. Brands

**Affiliations:** 1 Department of Internal Medicine, AdventHealth Sebring, Sebring, FL, United States; 2 Department of Gastroenterology, Baylor College of Medicine, Houston, TX, United States; 3 University of Columbia, New York, NY, United States; 4 Department of Gastroenterology, AdventHealth Sebring, Sebring, FL, United States

**Keywords:** area health resource files, CDC WONDER, epidemiology, GIPD, mortality rate, upper gastrointestinal bleeding

## Abstract

**Background:**

Upper gastrointestinal bleeding (UGIB) continues to pose a significant burden on the national healthcare system. However, geographical patterns of UGIB-associated mortality have not been explored in depth. Therefore, we investigated trends of UGIB mortality in the United States and examined the impact of primary care provider density (PCPD) and gastrointestinal physician density (GIPD).

**Methods:**

ICD-10 codes for UGIB mortality were identified using the CDC WONDER database. Crude mortality rates (CMRs) and age-adjusted mortality rates (AAMRs) were extracted from the Multiple Cause of Death files covering 1999–2023 using the 2000 US Census as the standard population. Correlation and linear regression analyses were performed to evaluate Area Health Resource File (AHRF) study variables, including, but not limited to, PCPD and GIPD.

**Results:**

The overall CMR for UGIB during the study period (1999–2023) was 12.1 per 100,000 population, whereas the AAMRs were 11.0 per 100,000 population in the United States. Overall mortality rates increased during the period 2018 to 2021 [annual percent change (APC): +9.05] and decreased from 2021 to 2023 (APC: −7.93). CMRs were highest among older age groups, American Indian or Alaska Native populations, and rural communities. Men had higher AAMRs than women. From 1999 to 2023, states that ranked in the top 90th percentile for overall CMRs included West Virginia, Rhode Island, Vermont, South Dakota, New Mexico, and Wyoming. According to data from AHRF, GIPD in the 50 states was significantly associated with CMRs, with the lowest quartiles of GIPD associated with the highest UGIB mortality [odds ratio (OR): 1.121, 95% confidence interval (1.090–1.153), *p* < 0.001]. In contrast, PCPD was not associated with CMRs (*p* = 0.120).

**Conclusion:**

GIPD, rather than PCPD, was significantly associated with higher CMRs for UGIB.

## Highlights

- *What is known*:
- Upper gastrointestinal bleeding (UGIB) continues to be a significant burden on the healthcare system in the United States.
- Sociodemographic factors influence UGIB mortality among certain highly vulnerable populations.
- *What is new here*:
- Overall mortality rates associated with UGIB increased during from 2018 to 2021.
- Gastrointestinal physician density (GIPD) was significantly associated with higher UGIB mortality rates in the majority of states, whereas primary care physician density (PCPD) showed no association with UGIB mortality.

## Introduction

Upper gastrointestinal bleeding (UGIB) is a potentially life-threatening condition that affects millions of individuals worldwide, according to Global Burden of Disease statistics (1). Its etiology includes multiple causes, such as peptic ulcers, esophageal varices, and carcinomas. UGIB accounts for nearly three-fourths of all causes of acute gastrointestinal (GI) bleeding (2) and is estimated to have an annual incidence of 80–150 per 100,000 population, with a case fatality rate ranging from approximately 2–15% (3). UGIB is defined as blood loss originating from the gastrointestinal tract above the ligament of Treitz and can manifest as hematemesis, hematochezia, and/or melena (3). In the United States, UGIB has previously been reported as a significant cause of morbidity and mortality, with an estimated mortality rate of approximately 10% (4–6).

To date, few studies have evaluated UGIB mortality rates with respect to demographic, geographic, and socioeconomic variability. One recent report demonstrated increased UGIB incidence and mortality in the United States following the onset of the coronavirus disease 2019 (COVID-19) pandemic (7). However, the currently available literature has not examined the mortality rate associated with UGIB in the United States over an extended period. Furthermore, the geographical distribution of UGIB-associated mortality has not been explored in depth. Therefore, we investigated trends in UGIB mortality in the United States across varying demographic, socioeconomic, and geographic patterns, including the density of primary care providers (PCPs) and gastrointestinal (GI) physicians at state and county levels.

## Materials and methods

This cross-sectional study utilized the de-identified publicly available data from the Centers for Disease Control and Prevention Wide-Ranging Online Data for Epidemiologic Research (CDC WONDER) database (8). The CDC WONDER database is highly regarded in healthcare policymaking because it uses the National Vitals Statistics Systems (NVSS), which is known for accurately recording over 99% of deaths that occur in the United States, regardless of the cause, whether known or unknown (9). The Institutional Review Board (IRB) of AdventHealth determined on 29 February 2024 that this research proposal did not require informed consent from study participants. IRB approval was granted on 4 March 2024 to commence the study (IRB number: 2165646–2). The study findings adhered to the reporting guidelines of the STROBE statement (10) and were conducted in compliance with the ethical standards set by the Declaration of Helsinki and the responsible institution regarding human subject determination.

Mortality caused by UGIB was identified by applying the International Classification of Diseases, Tenth Revision (ICD-10) codes listed in Supplementary Table 1. Variables including sex, age group, race, US census region, urbanization, and state and county of residence were abstracted from the Multiple Causes of Death files, which reported crude mortality rates (CMRs) and age-adjusted mortality rates (AAMRs). Age-standardized mortality rates were estimated using the automated predefined method provided by CDC WONDER, using the 2000 US Census as the standard population, and were reported per 100,000 population (11, 12). All age groups reporting mortality were included without restriction. The study period was selected as 1999–2023 because of annual data availability, using the 2000 US Census as the standard population. Data from 2024 were excluded because they were incomplete at the time of study commencement. The analysis focused on specific variables described in detail in Supplementary methods.

The primary objective of the study was to investigate trends in UGIB mortality across varying demographic and geographic factors and to determine whether PCPD and gastrointestinal physician density (GIPD) significantly impacted UGIB mortality rates in the United States. To achieve this objective, Area Health Resource Files (AHRFs) from the Health Resources and Services Administration (HRSA) were utilized. The most recent available files were from 2021 and were released during 2022–2023 (13). The total physician workforce including both MDs and DOs was used as a descriptive measure for each state, and the number of PCPs and GI physicians in each county across all 50 states was extracted. PCPD and GIPD were subsequently calculated per 100,000 population using county-level data. Additional variables, including median age, total population from the 2020 US Census, annual per capita income, and annual household income, were also obtained from AHRF to characterize sociodemographic and socioeconomic factors across geographical regions. A detailed methodological description of these variables is provided in Supplementary methods.

Spearman’s correlation (rho) and linear regression (β coefficients) analyses were performed to evaluate AHRF study variables, including PCPD and GIPD, to assess mortality trends and quantify associations with UGIB-related mortality in geographically vulnerable populations. Associations between GIPD quartiles and UGIB-related mortality were assessed by calculating mean differences with standard deviation for each quartile, along with medians and interquartile ranges, followed by application of the Kruskal–Wallis *H* test to determine statistical significance. A two-tailed *p*-value of 0.05 was considered statistically significant. To improve robustness, sensitivity analyses were also performed by setting the UGIB crude mortality rate as the dependent variable and calculating state-specific correlations using county-level data. A total of 3,012 counties were included in the final analysis after the exclusion of 137 counties because of missing data. Reasons for missing data in these counties and their corresponding estimated population according to the 2020 US census and AHRF 2021 are listed in Supplementary Table 2.

Statistical analyses were performed using the CDC WONDER database for age standardization and adjustment, whereas Microsoft Excel Office 365 was used for data cleaning and management. Modeling analyses were initially conducted using Joinpoint Regression Program software developed by the National Institutes of Health (NIH), National Cancer Institute (NCI), Surveillance Research Program, Division of Cancer Control and Population Sciences, Bethesda, MD (version 5.0.2). Results were expressed as annual percent changes (APCs), average annual percentage changes (AAPCs), and 95% confidence intervals (CIs). Additionally, AHRF data for PCPD and GIPD were analyzed using the Statistical Package for the Social Sciences (SPSS; IBM Corp., version 25.0) to perform Spearman’s correlation and linear regression analyses, as appropriate. Furthermore, map charts were created using MapChart.net (14), and the Flourish application website (app.flourish.studio) (15) was used for data visualization and storytelling. These tools were used to generate state- and county-level mortality maps. The Flourish application was also used to generate network keyword maps and radial tree representations of region-wise state-level data, whereas grouped scatter plots for county-level data were generated using SPSS.

## Results

**Figure 1:**
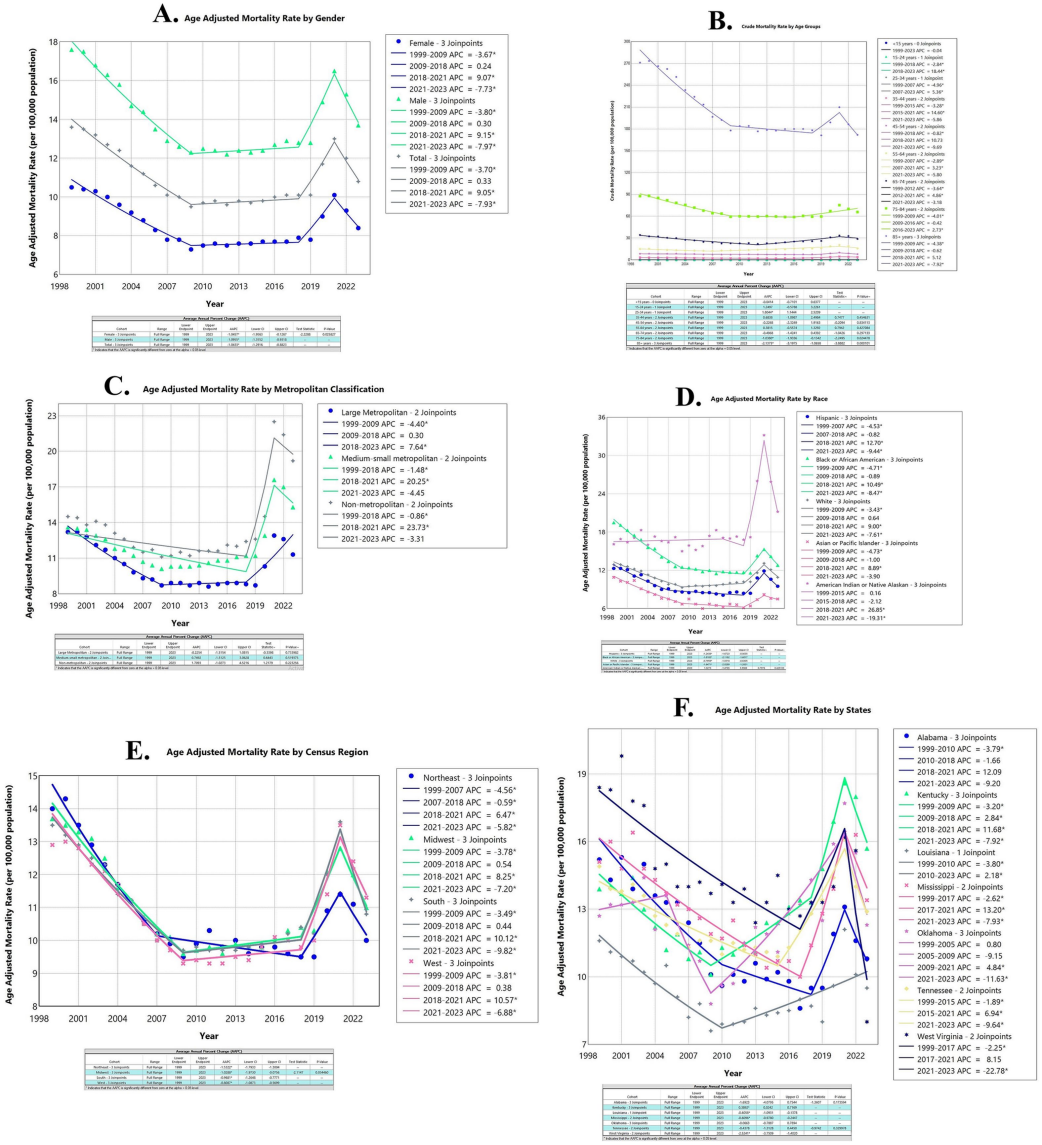
Age-adjusted mortality rate (AAMR) trends according to demographic and geographic variables: **(A)** sex, **(B)** age groups, **(C)** race, **(D)** urban–rural classification, **(E)** US census region, **(F)** states representing various percentiles of crude mortality rates.

Table 1 demonstrates the overall mortality rate from UGIB and correlations between mortality rates according to age groups, sex, race, US census region, and urbanization status using the 2013 National Center for Health Statistics (NCHS) Urban–Rural Classification Scheme for Counties (16). The overall CMRs for UGIB during the study period (1999–2023) were 12.1 per 100,000 population, whereas AAMRs were 11.0 per 100,000 population in the United States. Males had higher AAMRs than females (*p* = 0.025) (Figure 1A). Overall mortality trends increased during the period from 2018 to 2021 [annual percent change (APC): +9.05] and decreased from 2021 to 2023 (APC: −7.93).

**Table 1:** Stratification of crude mortality rates of upper gastrointestinal bleeding (UGIB) according to demographic variables among the US population (1999–2023).

Year	Deaths	Population cumulative, 1999–2023	Latest population (from CDC WONDER)	Relative percentages (from total deaths)	Crude mortality rate	Lower 95% confidence interval	Upper 95% confidence interval
Total: 1999–2023	939,121	7,744,825,506	333,287,557	100%	12.13	12.0	12.2
Gender							
Male	508,942	3,812,304,691	165,283,553	54.2%	13.35	13.3	13.4
Female	428,793	3,932,520,815	168,004,004	45.8%	10.90	10.8	11.0
Race							
Hispanic	71,042	1,267,256,074	63,664,346	7.6%	5.61	5.5	5.7
American Indian and Alaskan Native	9,507	63,504,100	2,420,972	1.0%	14.97	14.1	15.8
Asian and Pacific Islander	24,821	416,889,054	20,911,953	2.7%	5.95	5.6	6.3
Black or African American	110,370	989,931,288	42,070,471	11.8%	11.15	11.0	11.3
White	720,197	4,983,466,621	196,225,966	76.9%	14.45	14.4	14.5
Age group (years)							
<15	1,238	1,516,148,880	59,437,387	0.13%	0.08	0.05	0.1
15–24	1,441	1,067,566,527	44,341,571	0.15%	0.13	0.1	0.2
25–34	7,955	1,056,587,174	45,501,300	0.85%	0.75	0.7	0.8
35–44	30,967	1,062,081,872	43,695,365	3.3%	2.91	2.8	3.0
45–54	84,289	1,049,127,946	40,431,645	9.0%	8.03	8.0	8.1
55–64	134,220	893,398,785	42,085,437	14.3%	15.02	14.9	15.1
65–74	165,902	611,701,341	33,788,439	17.7%	27.12	27.0	27.2
75–84	236,686	349,751,598	17,520,545	25.2%	67.67	67.6	67.7
85+	276,607	138,461,383	6,485,868	29.4%	199.77	198.0	201.0
Census region							
Northeast	182,698	1,384,235,572	57,040,406	19.4%	13.19	13.1	13.3
Midwest	212,939	1,672,537,848	68,787,595	22.7%	12.73	12.7	12.8
South	343,250	2,882,475,794	128,716,192	36.5%	12.91	12.8	13.0
West	200,462	1,805,576,292	78,743,364	21.3%	11.10	11.0	11.2
*Urbanization							
Large, fringe Metropolitan	444,056	4,274,728,285	185,072,770	47.3%	10.39	10.3	10.5
Medium–small metropolitan	307,582	2,318,036,826	98,666,383	32.7%	13.27	13.2	13.3
Non-metropolitan	187,711	1,144,798,639	45,922,199	20.0%	16.40	16.3	16.5

*Large central metropolitan + large fringe metropolitan; medium metropolitan + small metropolitan; micropolitan + non-core = non-metropolitan. CDC, Centers for Disease Control and Prevention; CI, confidence interval; UGIB, upper gastrointestinal bleeding.

**Figure 2:**
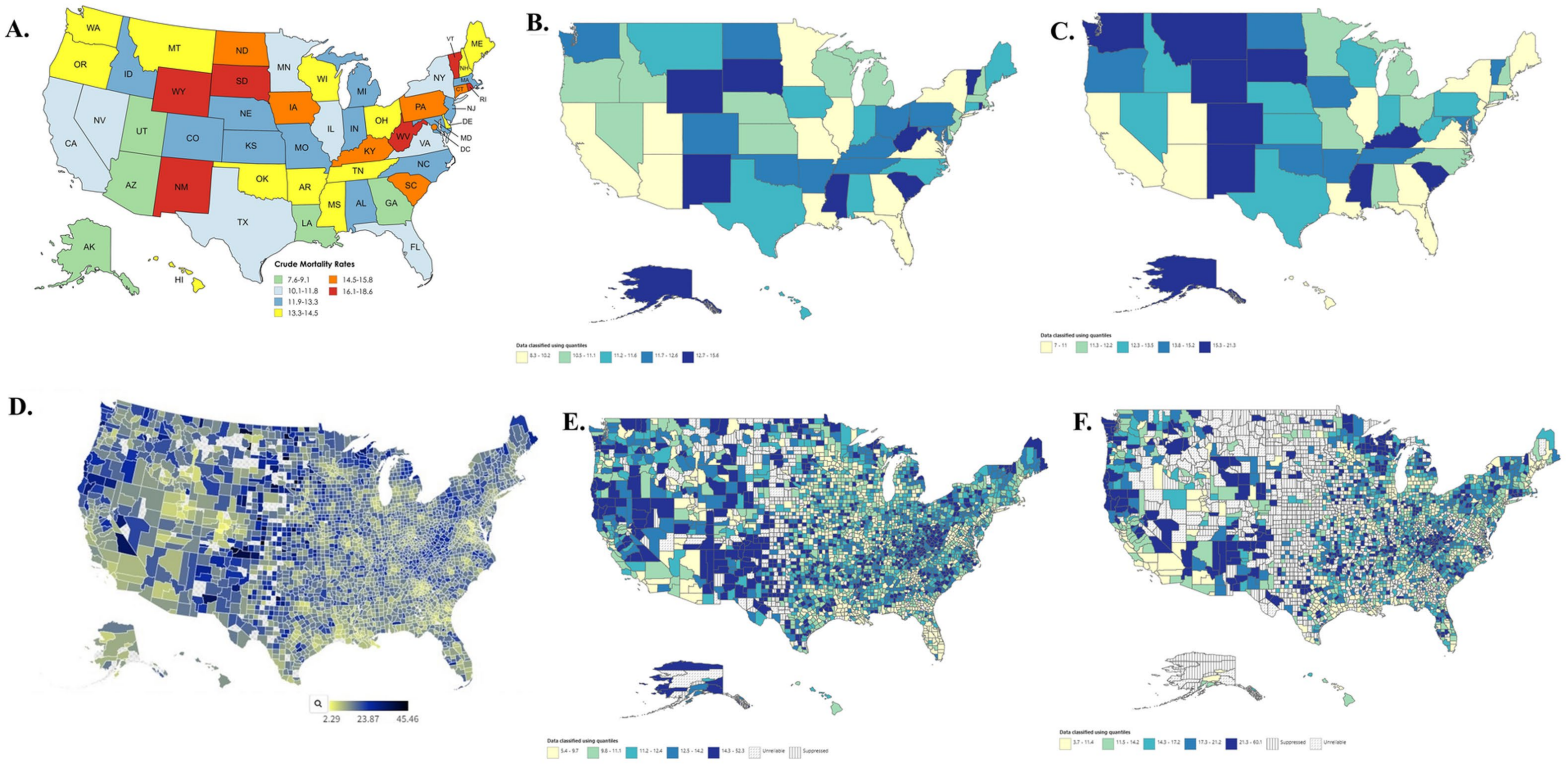
State- and county-wide distribution of mortality rates (crude and age-adjusted). **(A)** Crude mortality rates for all states (1999–2023). **(B)** Age-adjusted mortality rates for all states (1999–2020). **(C)** Age-adjusted mortality rates for all states (2021–2023). **(D)** Crude mortality rates for all counties (1999–2023). **(E)** Age-adjusted mortality rates for all counties (1999–2020). **(F)** Crude mortality rates for all counties (2021–2023).

Crude mortality trends for all study variables from 1999 to 2023 are presented in Supplementary Tables 3, 4. CMRs were highest among older age groups (Figure 1B), whereas AAMRs were highest in rural communities (Figure 1C) and among American Indian or Alaska Native populations (Figure 1D), but did not differ substantially across US census regions (Figure 1E). States within the top 90th percentile for overall CMRs from 1999 to 2023 included West Virginia, Rhode Island, Vermont, South Dakota, New Mexico, and Wyoming (Figures 2A–C).

Seven states were additionally selected to demonstrate varying AAMR trends across both high- and low-mortality percentiles. These included West Virginia (100th percentile), Kentucky (76th percentile), Oklahoma (66th percentile), Mississippi (64th percentile), Tennessee (60th percentile), Alabama (42nd percentile), and Louisiana (4th percentile), as shown in Figure 1F. Figures 2D–F further illustrates county-level CMRs and AAMRs using CDC WONDER map graphs. State-level AAMRs for 1999–2020 and the more recent period of 2021–2023 are separately presented in Supplementary Table 4. Furthermore, the top 90th percentile states were stratified according to the 2013 NCHS Urban–Rural Classification Scheme, and the most represented states within urban, suburban, and rural community belts were identified (Supplementary Table 5).

According to AHRF data, as presented in Table 2 and Supplementary Figure 1, PCPD and GIPD were calculated from AHRF data to perform logistic regression analyses using county-level data stratified by state. GIPD [OR: 1.121, 95% CI: (1.090–1.153), *p* < 0.001], but not PCPD (*p* = 0.120), was significantly associated with higher CMRs of UGIB across most of the 50 states. This association remained significant after adjustment for demographic and socioeconomic factors, including median age, total census population, per capita income, and annual household income, as shown in Table 3 (*p* < 0.001).

**Table 2:** State-wide characteristics of area health resource files (AHRF) datasets with CDC crude mortality data.

No.	State	No. of counties included in the crude analysis	Total population (census 2020 from AHRF)	Total deaths (1999–2023)	Crude mortality rate per 100,000 population (1999–2023)	Total physician workforce (AHRF)	Primary care physicians (AHRF)	GI physicians (AHRF)
AL	Alabama	67	4,997,675	17,345	12.98	11,961	3,208	186
AK	Alaska	17	735,951	1,984	9.89	2,114	709	11
AZ	Arizona	15	7,079,203	18,279	9.92	17,347	4,835	303
AR	Arkansas	75	3,006,310	11,825	14.34	6,491	2,047	98
CA	California	57	39,455,353	107,225	10.15	110,826	31,820	1754
CO	Colorado	57	5,723,176	17,960	12.01	18,322	4,816	222
CT	Connecticut	8	3,605,330	14,574	14.61	14,058	2,983	294
DE	Delaware	3	981,892	3,441	13.31	3,262	740	39
DC	District of Columbia	1	683,154	2,594	15.05	6,976	863	77
FL	Florida	67	21,339,762	65,468	11.83	61,273	15,903	1,109
GA	Georgia	153	10,625,615	26,065	9.41	27,117	7,121	411
HI	Hawaii	4	1,453,498	5,302	13.91	4,999	1,285	55
ID	Idaho	41	1,811,617	5,465	11.88	3,251	1,174	47
IL	Illinois	102	12,821,813	38,195	10.74	40,556	10,026	596
IN	Indiana	92	6,751,340	24,277	12.93	15,649	4,465	233
IA	Iowa	99	3,179,090	13,113	14.97	7,981	2,305	82
KS	Kansas	100	2,932,099	10,471	12.92	6,220	2,284	99
KY	Kentucky	119	4,494,141	18,170	14.56	10,006	2,816	182
LA	Louisiana	63	4,657,305	12,493	9.70	11,800	3,210	240
ME	Maine	16	1,357,046	5,271	14.39	4,104	1,473	49
MD	Maryland	24	6,148,545	21,763	13.26	22,851	5,227	366
MA	Massachusetts	13	6,991,854	24,742	13.24	33,125	7,053	596
MI	Michigan	83	10,062,512	36,160	12.86	33,879	7,875	370
MN	Minnesota	86	5,670,472	18,118	11.75	16,413	5,033	296
MS	Mississippi	81	2,967,023	11,622	13.94	5,900	1,573	113
MO	Missouri	114	6,141,534	20,550	12.15	17,871	4,342	275
MT	Montana	43	1,077,978	4,195	14.53	3,043	920	36
NE	Nebraska	73	1,951,480	6,621	12.54	4,955	1,462	73
NV	Nevada	14	3,059,238	8,569	11.13	6,201	1,745	87
NH	New Hampshire	10	1,372,174	4,998	13.29	4,841	1,209	73
NJ	New Jersey	21	9,234,024	32,375	12.98	29,402	7,245	591
NM	New Mexico	31	2,109,366	9,346	16.23	5,333	1,574	67
NY	New York	62	20,114,745	59,804	10.95	87,943	15,938	1,535
NC	North Carolina	100	10,367,011	32,793	12.15	28,305	7,469	525
ND	North Dakota	46	773,344	3,067	15.38	1,700	599	15
OH	Ohio	88	11,769,923	45,745	14.15	37,504	8,868	529
OK	Oklahoma	76	3,948,136	15,095	13.94	9,477	2,354	113
OR	Oregon	33	4,207,177	15,184	13.75	14,242	3,989	181
PA	Pennsylvania	67	12,970,650	56,093	15.79	46,623	10,252	761
RI	Rhode Island	5	1,091,949	5,048	16.82	4,443	1,060	83
SC	South Carolina	46	5,078,914	19,997	14.77	14,699	3,495	191
SD	South Dakota	54	881,785	3,959	16.71	2,650	715	26
TN	Tennessee	95	6,859,497	25,550	13.89	19,410	4,854	299
TX	Texas	233	28,862,581	76,149	10.32	71,063	17,818	1,097
UT	Utah	25	3,231,370	6,203	7.64	8,072	1,870	107
VT	Vermont	14	641,637	2,973	16.77	2,213	718	33
VA	Virginia	132	8,582,479	23,827	10.48	23,222	6,443	387
WA	Washington	38	7,617,364	26,911	13.70	23,538	6,440	340
WV	West Virginia	55	1,801,049	9,493	18.60	5,001	1,352	68
WI	Wisconsin	72	5,871,661	21,668	13.27	16,071	4,713	218
WY	Wyoming	23	576,641	2,592	16.11	1,248	402	13
All United States combined		3,012	329,725,483	1,070,727	12.13	985,575	248,730	15,551

Counties were excluded if crude mortality rates were not reported (suppressed or unreliable if fewer than 10 according to CDC WONDER guidelines). AHRF, Area Health Resource Files; CDC, Centers for Disease Control and Prevention; GI, gastrointestinal.

**Table 3:** Regression analysis of CDC WONDER crude mortality rates adjusted for area health resource files (AHRF) variables.

No.	Independent variables	Reference: GI physician density (4th quartile)	GI physician density (2nd–3rd quartile)			GI physician density (1st quartile)		
			Odds ratio	95% confidence interval	*p*-value	Odds ratio	95% confidence interval	*P*-value
1	CDC WONDER upper GI bleed (crude mortality rate)	–	1.036	0.997–1.078	0.071	1.121	1.090–1.153	<0.001^*^
2	Adjusted by median age	–	1.025	0.983–1.069	0.253	1.083	1.050–1.116	<0.001^*^
3	Adjusted by total population (census)	–	1.016	0.977–1.057	0.423	1.038	1.007–1.070	0.016^*^
4	Adjusted by per capita income	–	1.010	0.973–1.048	0.601	1.085	1.055–1.115	<0.001^*^
5	Adjusted by annual household income	–	1.006	0.967–1.046	0.774	1.039	1.009–1.070	0.010^*^
6	Adjusted by all of the above except PCPD (AHRF)	–	0.991	0.947–1.037	0.698	0.991	0.958–1.025	0.591
7	Further adjusted by PCPD (AHRF)	–	1.046	1.005–1.088	0.027^*^	1.146	1.109–1.183	<0.001^*^
8	Adjusted by all of the above, including PCPD (AHRF)	–	1.020	0.969–1.073	0.452	1.043	1.001–1.087	0.048^*^

*Indicates significant *p*-values. AHRF, Area Health Resource Files; CDC, Centers for Disease Control and Prevention; GI, gastrointestinal; PCPD, primary care physician density.

Lower quartiles of GIPD demonstrated a highly significant association with increasing CMRs (*p* < 0.001), as shown in Table 4 and Supplementary Figure 2. Supplementary Figures 3, 4 further illustrate census region-, state-, and county-level analyses of CMRs and GIPD. The methodology used to determine GIPD quartiles is described in detail in Supplementary methods.

**Table 4:** States in four quartiles of GI physician density.

	1st quartile (<3.41 per 100,000)	2nd and 3rd quartile (3.41–5.10 per 100,000)		4th quartile (>5.10 per 100,000)
	AK	AL	AZ	CT
	AR	CO	CA	DC
	IA	GA	DE	FL
	ID	HI	IL	LA
	KS	IN	KY	MA
	MT	ME	MO	MD
	ND	MI	NC	MN
	NM	MS	OH	NH
	NV	NE	OR	NJ
	OK	SC	TN	NY
	SD	TX	VA	PA
	UT	WI	WA	RI
	WY	WV	-	VT
Mean crude mortality rate	15.56 ± 5.35	13.72 ± 4.22		13.04 ± 4.02
Median crude mortality rate	14.84 (12.03–18.36)	13.57 (10.73–16.52)		12.78 (10.18–15.25)

GI, gastrointestinal. Kruskal–Wallis H was significant at the level of *p* < 0.001. 1st quartile, <25th percentile; 2nd and 3rd quartile, 25th–75th percentile; 4th quartile, >75th percentile.

## Discussion

The overarching reasons for the UGIB mortality trends noted in our study are likely multifactorial. The overall increase in mortality during 2018–2021 correlated with the onset and midpoint of the COVID-19 pandemic. Previous studies have reported increased mortality rates from UGIB during the COVID-19 pandemic, although the exact reasons remain unclear (17, 18). Corticosteroid use and the need for mechanical ventilation, which are often required in severe COVID-19 disease, are known risk factors for gastrointestinal bleeding (19, 20). Additionally, there were major disruptions in healthcare delivery systems during the pandemic, which reportedly caused delays in medical interventions (21–23). Furthermore, physicians may have experienced increased patient congestion during the pandemic, limiting their ability to provide adequate care to underserved populations, particularly those with high social vulnerability indices. Limited access to healthcare, especially in underserved areas, may therefore have contributed to delayed care and increased mortality.

Anticoagulation is a known contributor to GI bleeding, and increased coronary interventions may be related to increased anticoagulation use. Although the Lown Institute reported that 229 unnecessary stents were placed during 2019–2021, accounting for approximately 22% of all stents placed during that period, there was no indication whether the number of stents placed was higher compared with previous years. Therefore, it remains unclear whether anticoagulation use increased during this period (24). Prehospitalization non-steroidal anti-inflammatory drug (NSAID) use was also common during the COVID-19 pandemic for symptom relief (25).

NSAIDs are known to cause mucosal injury through inhibition of cyclooxygenase-1 (COX-1), resulting in reduced cytoprotective mucosal prostaglandins and decreased bicarbonate secretion, which serves as a protective mucus barrier in the stomach and small bowel (26).

The influence of COVID-19 infection on UGIB mortality rates has been widely reported and has previously been a topic of significant interest among researchers worldwide (7). However, the current study provides valuable insights into the impact of UGIB mortality over an extended period beyond the pandemic, including various geographic, demographic, and socioeconomic factors in the United States population at the county level. Studies have shown that alcohol sales increased during COVID-19, which might have contributed to increased mortality from UGIB (27, 28). The annual average prevalence of tobacco use in West Virginia during 2017–2019 was 39.7%, which was higher than both the regional and national averages of 28.2 and 26.8%, respectively. This finding mirrors the 100th percentile AAMRs observed for West Virginia in our study and may represent one of the contributing factors (29).

Studies have also shown that, in general, American Indians and Alaskan Natives have poorer health outcomes. For instance, COVID-19 infection rates were reported to be 3.5 times higher among American Indians/Alaska Natives than among non-Hispanic white individuals. This observation agrees with the trend of the highest AAMRs noted among American Indians/Alaska Natives in our study (30). Of note, states with larger American Indian/Alaska Native populations include New Mexico and South Dakota, both of which also had higher UGIB-associated AAMRs (31).

We observed that the mortality rate increased, especially in geographical areas where GI physicians were less dense. However, the State of Utah, despite being in the first quartile (less than 25th percentile) of GIPD, was found to have the lowest crude rate (7.64 per 100,000) and one of the lowest AAMRs nationwide. The reason for this negative correlation is unclear. Interestingly, the life expectancy in Utah is 1.6 years higher than the life expectancy in the United States overall. Utah overall and especially Provo County in Utah is reported to have ideals promoted in blue zones (32, 33).

The other exception was Rhode Island, despite being in the highest fourth quartile (above the 75th percentile) of GIPD, which was found to have high CMRs as well as no significant correlation on Spearman’s metrics within the two parameters. Rhode Island (RI) has a higher poverty rate compared to the entire US (34). RI is noted to have higher excessive drinking compared to the overall US rates (35). Moreover, chronic liver disease and cirrhosis are listed as the 9th leading cause of death in RI. Therefore, the risk of variceal bleeding is likely to increase in the setting of cirrhosis (36). These factors are possible contributors to the higher CMRs noted in RI.

We further reviewed the literature for similar trends and findings from studies of UGIB; some of those evaluated the mortality and case fatality rates. In Finland, it was found to be 1.3% of UGIB, with an age-standardized incidence of UGIB of 0.94 per 1,000 participants (94/100,000). Similar to our results, they had higher mortality in males than in females by about a ratio of 5–10% (37). One study from Valencia, Spain, observed the use of non-steroidal anti-inflammatory drugs (NSAIDs) by monitoring the pharmacovigilance (trends in sales) with the trends of UGIB in the same region (38). They found that an increase in NSAID use in that period (2000–2005) was associated with an increase in the incidence of UGIB (although mortality was not reported). However, the trends did not show an increase in UGIB hospitalization rates. A very small-scale study from Syria from 2018 to 2020, on the other hand, showed findings similar to our study, with a mortality rate reportedly of 9.4% (39). More recently published population-based studies demonstrated an increase in hospitalization and mortality related to UGIB (40, 41). Some of the factors reportedly demonstrated a rise in the use of anti-inflammatory drugs, steroids, and anti-coagulants, as well as an aging population, as the main contributing factor to recent increases in mortality of UGIB (42). However, a few studies negated these ongoing trends (43, 44). Our study revealed that mortality from UGIB increased with increasing age. This could be related to higher preexisting comorbidities in this age group, which predispose them to overall mortality.

Various demographic determinants could contribute to the geographical variations in UGIB mortality rates for certain states, with increased trends, such as Wyoming, West Virginia, and notably Kentucky, with an AAPC of 0.39 (95% CI: 0.02–0.72), as shown in Figure 1F. These factors might include median age, lifestyle, socioeconomic status, and environmental factors. Some of them were reproducible in our study. For instance, median age and annual household income were found to be the most significant predictors of increased mortality rates across most United States populations. Other modifiable factors that might influence the current study’s outcome were the lack of GI physicians in most of the rural communities of these underserved states, examples of which can be observed in Table 4 and Supplementary Table 5.

Some of these factors are directly attributed to low funding for healthcare talent pipeline programs, including graduate medical education, and their inequitable distribution across the United States. More alarming with these trends are worsening access to healthcare services, resulting in delayed diagnosis and management, and the overburdening and strain on healthcare systems, especially the scarcity of GI physicians. Additionally, this study highlighted the urgent need for targeted interventions and public health measures to mitigate the increased burden faced by the already burdened GI physicians’ workforce nationally, with a mean number of 4.41 +/− 1.77 SD per 100,000 population on state-level datasets. These data further emphasized the importance of educating and equipping more gastroenterologists with masterful expertise and skills in evaluating complex patients and performing endoscopic procedures. Gastroenterologists are critical to the provision of timely direct medical care and evidence-based consultation for prompt interventions and continued monitoring post-intervention by focusing on long-term outcomes to prevent and decrease mortality from UGIB and other life-threatening conditions.

Overall, the current study represents a significant addition to our understanding of the medical and public health implications of the UGIB and underscores the importance of continuous ongoing epidemiological research and data analysis to guide effective interventions and policymaking decisions (45, 46). Based on the AAMC Physician Specialty Data Report of 2022, the number of actively practicing gastroenterologists is 15,678, while the number of patients per gastroenterologist is 20,830 (47); this data point underscores a growing national healthcare crisis and a significant physician specialists’ shortage in gastroenterology. Most expert consensus over the last several decades reflects that such predictions are often underestimates of the true public health needs, particularly in rural non-metropolitan areas.

Furthermore, according to the physician workforce data published by AAMC, as of 2023, the states of South Dakota, Vermont, and Wyoming each had 3, 4, and 2 gastroenterologists per 100,000 population, respectively (48). According to data from the American College of Gastroenterologists (ACG), there are zero (0) gastroenterology fellowships in the state of South Dakota, one in Vermont and zero in Wyoming (49). Notably, in our study, these three states are among the states with the highest AAMRs from UGIB.

This study has several strengths. To our knowledge, the current study is the most comprehensive analysis of the national mortality trends based on demographic (gender, age groups, racial differences), socioeconomic (such as per capita income and household income), regional and geographical factors (such as US census region, urbanization, and state to county-level data) by using unadjusted and adjusted estimates for UGIB as the underlying cause of mortality. This data, as we understand, is the first ever reported in-depth analysis that explores mortality, and is correlated with the density of GI Physicians, and the findings can be a potential landmark data to serve as a springboard to action to strengthen the GI Physician workforce.

Our analysis has several limitations. First, the reliance on data from death certificates, which can contain inaccuracies and may not always provide a complete picture of the patient’s medical history on the CDC WONDER platform, is a non-modifiable risk of bias. Methodologically, reliance solely on death certificate data from CDC WONDER without inclusion of UGIB incidence or prevalence data limits the interpretation of mortality drivers. The potential biases and confounding factors arise, including potential underlying causes of death. Because we included patients with UGIB having multiple causes of death listed on their death certificates, UGIB may have been the reason for admission and therefore contributed to mortality, but it may or may not be the underlying cause of death. Moreover, the analysis based on death certificates does not always connect or list the other risk factors contributing to mortality. Third, another area for improvement is that the study only examined mortality rates and did not analyze the incidence and prevalence of UGIB in those vulnerable geographical areas for in-depth analysis, which could provide a more comprehensive understanding of the issues regionally. The prevalence of the disease might only be the single most compelling risk factor in a certain region, population, or race for determining a high mortality rate, which was missing in the current study and might provide fruitful ground for future studies to take place. Additionally, the study did not focus on the impact of specific risk factors on UGIB, such as medication use or underlying medical conditions, the data for which were not available in both the resources used in this study, i.e., CDC WONDER and AHRF. For CDC WONDER specifically, the data from 2022 to 2023 is still reported provisionally and cannot be relied upon, although we can expect only minor changes once they become finalized published reports in the next couple of years. Furthermore, the physician density captured on AHRF may not be updated, and as such, some of the physicians included in the density may have relocated.

Finally, these data may not be representative of the entire population or may not capture changes over time or differences across subgroups accurately due to missing data, which can limit the generalizability of the study findings (9). Since the CDC WONDER database only provides information on mortality rather than on morbidity or other outcomes of interest, this may limit the scope of the study. For the AHRF study files, the 2021 datasets were used as they were the most recent dataset files available on the AHRF portal. However, we, as abstracting authors, do not see any major discrepancy in the data reported over the years with recent updates of provisionally reported data. Lastly, the observed association between GIPD and UGIB mortality is weak due to a non-monotonic relationship and attenuates in multivariable models, with re-emergence only upon including PCP density in the model, suggesting collinearity and that GIPD may function primarily as a proxy for broader healthcare system capacity and does not accurately represent expansion of functional access to emergent endoscopic care, but focuses on reframing geographic disparities in UGIB outcomes and healthcare resource distribution.

## Conclusion

The study highlights the need for ongoing research correlating publicly available databases to strengthen the understanding of the underlying mechanisms and sociodemographic and geographical factors associated with UGIB. Such findings may help better inform healthcare policy and clinical decision-making. Finally, fewer hospitals, limited resources, decreasing availability of GI Physicians, scarcity of well-developed endoscopic suites in underdeveloped rural areas, and lack of medical education programs are likely factors contributing to the findings in our study.

It is of paramount importance that hospitals, governmental agencies, ACGME programs, and physicians work together to mitigate mortality from UGIB. We strongly recommend improving infrastructure, providing additional resources for GME programs, and establishing more widely and optimally distributed fellowship programs in areas with low GIPD to effectively address nationwide healthcare workforce needs, including GIPD. Patients require a knowledgeable, skilled, well-equipped GI physician workforce capable of responding promptly to UGIB emergencies and thereby preventing unnecessary morbidity and mortality from this condition and many others. This study represents findings that support a call to action to encourage internal medicine resident physicians to careers in gastroenterology and other workforce subspecialties needed before the crisis worsens in American healthcare. Residents, fellows, and attending physicians will need to be increasingly equipped to lead teams that can quickly and effectively address the complex needs of patients in limited-resource settings. Additionally, incentive programs should be implemented to encourage gastroenterologists to practice in highly vulnerable areas.

## Data Availability

The original contributions presented in the study are included in the article/Supplementary material, further inquiries can be directed to the corresponding author.
