# Supplementary material for: Critical shortage of gastrointestinal physician density in the USA: impact on mortality from upper gastrointestinal bleeding
Source: Front Med (Lausanne). 2026 Jun 15;13:1812471. doi: 10.3389/fmed.2026.1812471 (PMC13310767; doi:10.3389/fmed.2026.1812471)
Supplement: Supplementary file 1 [file Table_1.docx]

**Supplemental Material**

1. **Supplemental Methods**

***CDC WONDER***

In the CDC WONDER database, we accessed the Current Final Multiple Cause of Death Data for the years 1999-2000 and Provisional Multiple Cause of Death Data for the recent years i.e. 2021-2023. Both of these files were later merged for crude mortality rates after sorting out for differences. For age-adjusted mortality rates (AAMRs), a combined merger is not possible because of automated indirect methods by the CDC to utilize 2000 US age-standardized population [1] for age adjustment, and these coefficients are not available to us publicly. Hence, to avoid inaccuracies in data reporting, we would avoid reporting combined figures of AAMRs and would keep reporting figures dissected into 1999-2020 and 2021-2023 groups. An additional benefit of this segregation is that it will automatically allow us to follow trends from recent past years without any additional effort. For combined AAMRs in both datasets i.e., 1999-2020 and 2021-2023 (provisional), we have used indirect methods to calculate AAMRs from descriptions provided by CDC WONDER.

Age adjusted mortality rates is calculated by applying age-specific mortality rates (Ri) to the US standard population age distribution. The formula below is the method used by the National Center for Health Statistics (NCHS)

R' = S i ( Psi / Ps ) R i

Where

Psi = standard population for age group i

Ps = the total US standard population for all ages combined

The method for calculating confidence interval for 100 or more deaths is outlined below.

The lower 95% confidence interval (CI) = crude death rate + (1.96 x the standard error of the rate) => LCI = R - 1.96 * S (R)

The upper 95% confidence interval = crude death + (1.96 x the standard error of the rate) => UCI = R + 1.96 * S (R)

The method for calculating confidence interval for 100 or more deaths is outlined below.

The lower 95% confidence interval = crude death x lower 95% confidence limit factor for a death rate based on a Poisson variable of the number of deaths. LCI = R * L (0.95, D)

The upper 95% confidence interval = crude death rate multiplied by x the upper 95% confidence limit factor for a death rate based on a Poisson variable of the number of deaths. -> UCI = R * U (0.95, D)

Where:

LCI = lower 95% confidence interval

UCI = upper 95% confidence interval

R = crude death rate R = (deaths / population) * 100,000

D = the total number of deaths upon which the rate is based.

RSE(R) = relative standard error of rate RSE(R) = 100 * square root of (1/D)

S(R) = standard error of rate S(R) = R * (RSE(R) / 100)

Where:

R = crude death rate R = (deaths / population) * 100,000

D = the total number of deaths upon which the rate is based.

RSE(R) = relative standard error of rate RSE(R) = 100 * square root of (1/D)

After automated age-standardization from CDC WONDER tools [1], the data was abstracted for the above-mentioned years till December 2023, which is the current most updated year available on the database. However, this analysis has some limitations too. For instance, the data from 2022-2023 is still reported provisionally and cannot be relied upon completely till the figures are finalized currently by the CDC, although we can expect only minor changes since we recently saw finalized figures for 2022 and they appear to be in good shape and aligned with earlier years. Further, from provisionally reported data across the recent files (2021-2023), CDC WONDER has introduced some changes of features in the platform that allow differential categories like 6 races instead of regular 5 subdivisions and categorization into Occurrence and Residence at the time of death for data involving States and Counties. But overall, it didn’t skew the data much in our sensitivity analysis since the number of patients in the occurrence category at the time of death is negligible and more than 99.5% of that data has been reported in State of residence categories. So, despite some discrepancies in data of recent years (2021-2023) due to duplication of patients in occurrence and residence (for states and counties at the time of death), its overall effect on skewing the data is highly minimal.

For State-wide categorization of urbanization status, we further used sensitivity filters to make mutually exclusive groups in States for stratification into Urban and suburban (large central metropolitan + large fringe metropolitan), Suburban (Medium metropolitan + Small metropolitan) & Rural (Micropolitan + non-core = non-metropolitan) categories. This is unachievable to calculate AAMRs in the recent three years (2021-2023) because there are no AAMRs reported on county level or urbanization levels in these recent years, so only crude mortality data could be reported for certain variables. Also, Counties were excluded if they didn’t report crude mortality rates or AAMRs (suppressed or unreliable If the mortality figure less than 10 in number according to CDC WONDER guidelines). These were some of the notable limitations in navigating through the database. Although some reasoning was logical, like for age groups, we technically cannot extract AAMRs by definition, hence only crude mortality rates were reported for age groups. Age <15 was combined together as one subclass and similar 85+ was done too to be consistent. Age <15 had minimal mortality hence we couldn’t get any numbers other than suppressed or unreliable unless combining (merged) them as one group. For the rest of the age groups, a 10-year increment rule was applied for each group, rather than a narrowed 5-year group to avoid over-exploitation or avoidance of over analyzing crude mortality rates that were being reported.

***Area Health Resource Files (AHRF)***

We used the 2022-2023 version of AHRF datasets which are publishing most data gathered from AMA Physician Masterfile 2021; and Census County Population Estimates 2021. For the total population, we used the official AHRF census figure from 2020 rather than the estimated figures from 2021-2022 for reliability purposes. To calculate PCP density and GI physicians density, we divided the total number of physicians in each category by the total population for that particular State and/or County and multiplied it by 100,000 per population as standardized according to CDC WONDER mortality data. For median age, per capita income, and annual household income ($), we used the most recent available figures i.e., 2021. Also, when we were creating bar graphs to show median and interquartile range (IQR) for crude mortality rates (from CDC WONDER), and PCP density, GI physicians density, median age, per capita income, and annual household income from AHRF datasets, we only analyzed median values per State level for all counties represented (n=3012), this figure may actually differ from the cumulative sum for all total populations per State level.

***Files merger and data assimilation***

The final data merger was performed in Microsoft Excel Office version 365 before uploading it onto Joinpoint regression for trend model analysis. For quartile determination, we used BlueSKY Statistics version 10.0 owned by BlueSky Statistics, LLC, Chicago, IL, USA. Since only this feature was not available in SPSS version 25.0, the rest of all crude and inferential statistics were analyzed through the SPSS platform. For Spearman’s correlation, we eliminated introducing the risk of bias for null analysis by removing all 137 counties (See Supplemental Table 1) that either had no data reported or represented as zero crude mortality by either suppressed or unreliable figures within the CDC WONDER platform based on the regulation that no less than 9 deaths can ever be reported or published (in figures, graphs, maps, tables, etc.) in order to prevent attempts on learning the identity of any person or establishment included in these data. The concept of removing zero from dependent variables in Spearman’s correlation came from ‘correcting for bias in correlation coefficients due to intraindividual variability and non-normality’ [2]. Thus’ we determined it is better to remove zero values from the dependent variable i.e. crude mortality rates in this study to eliminate this risk of bias.

1. **Supplemental Figures**

Supplementary Figure 1. Box plots/bar graphs representing all included counties in each state for: (A) Crude mortality rate; (B) Median age; (C); Per capita income; (D) median household income; (E) Mean GIPD; (F) Mean PCPD.

**A.**


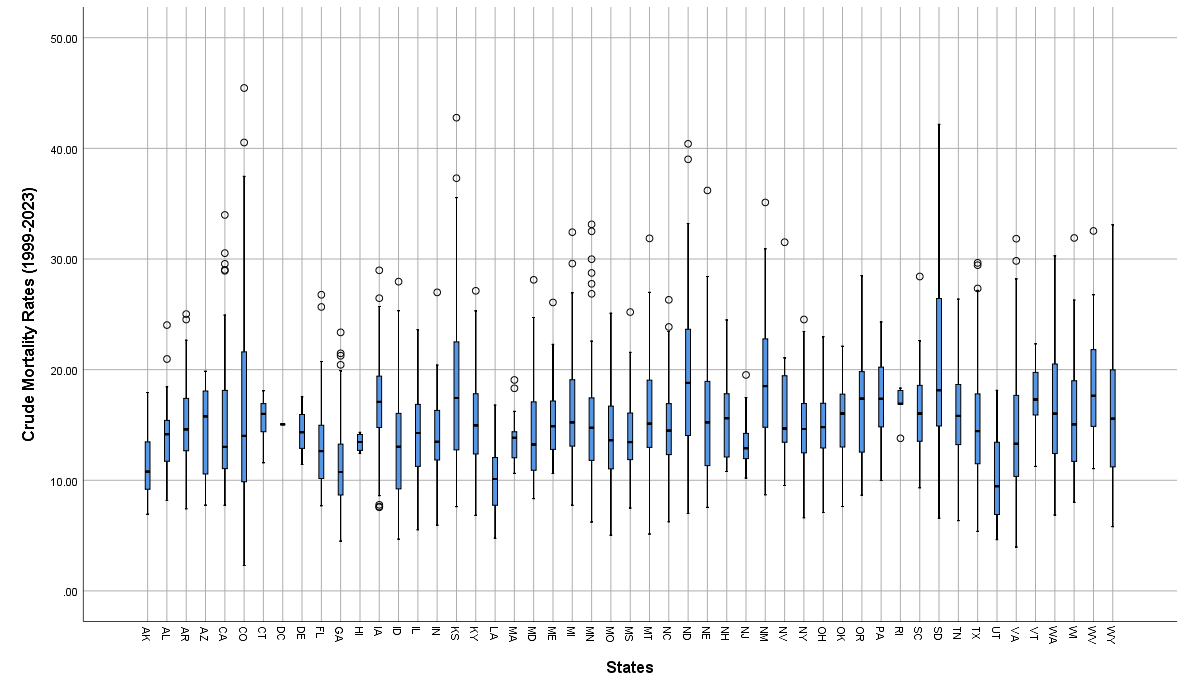


**B.**


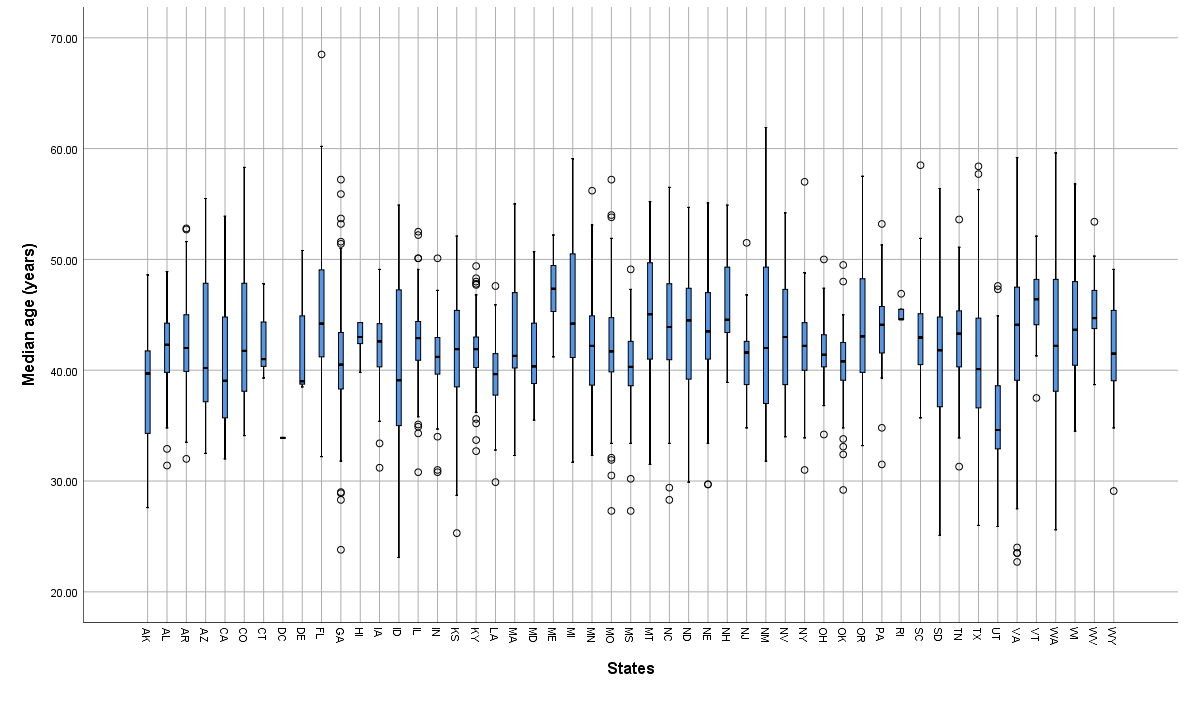


**C.**


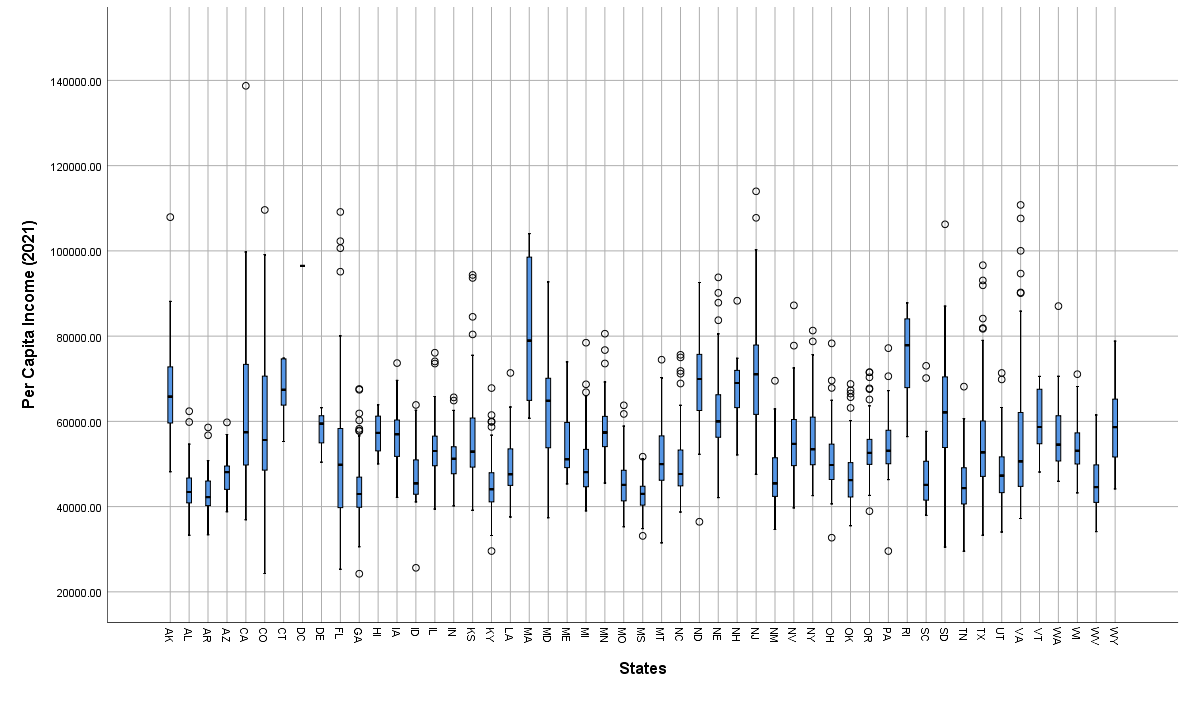


**D.**


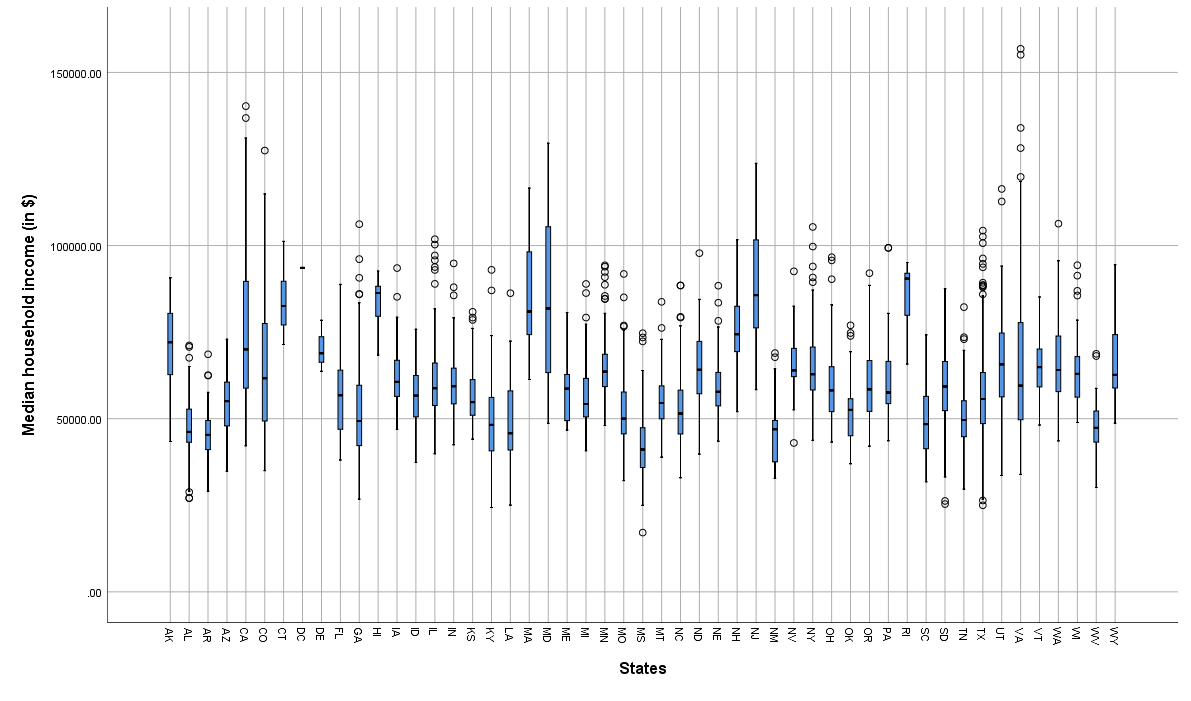


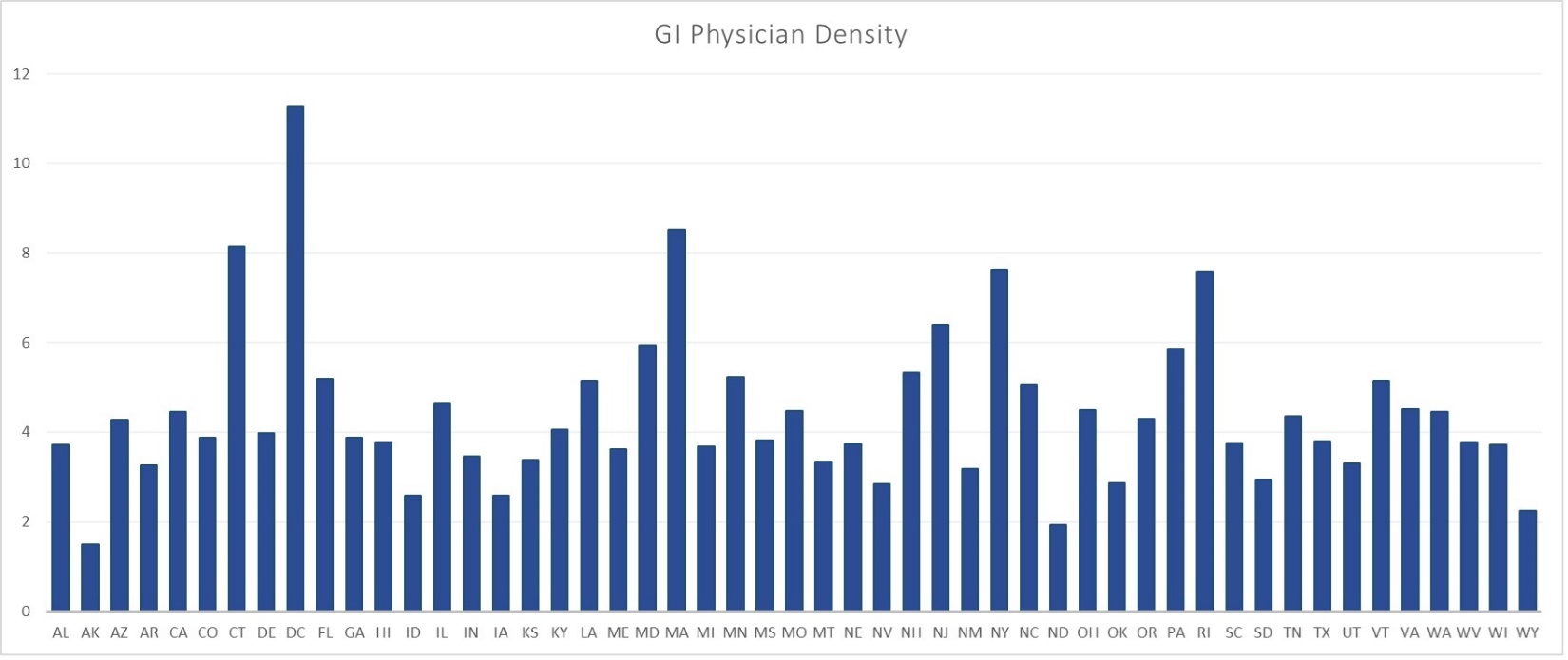
**E.**


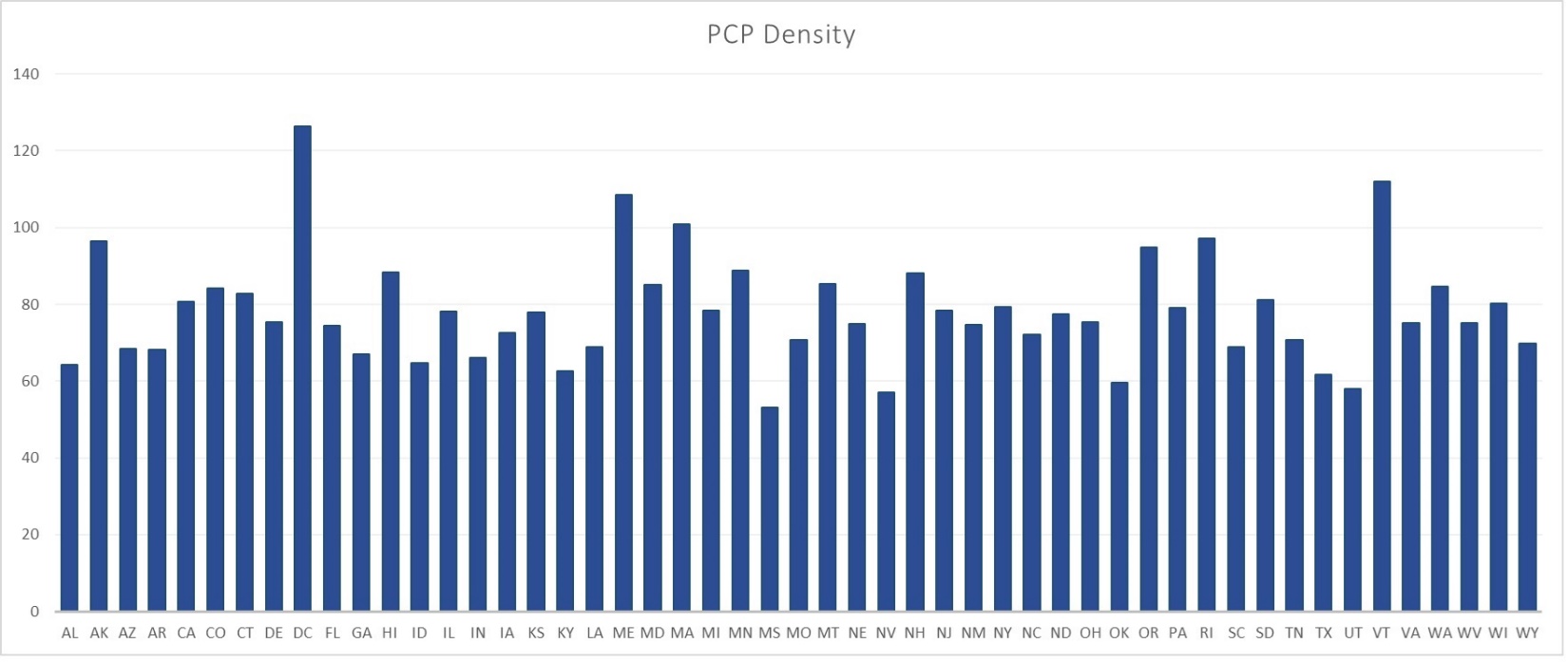
**F.**

**Supplementary Figure 2:** Keyword network map graph of all States representing crude mortality rates and of GI physicians density quartiles (Green = 1st quartile [<3.41], Blue = 2nd & 3rd quartile [3.41-5.10], Orange = 4th quartile [>5.10]).

**
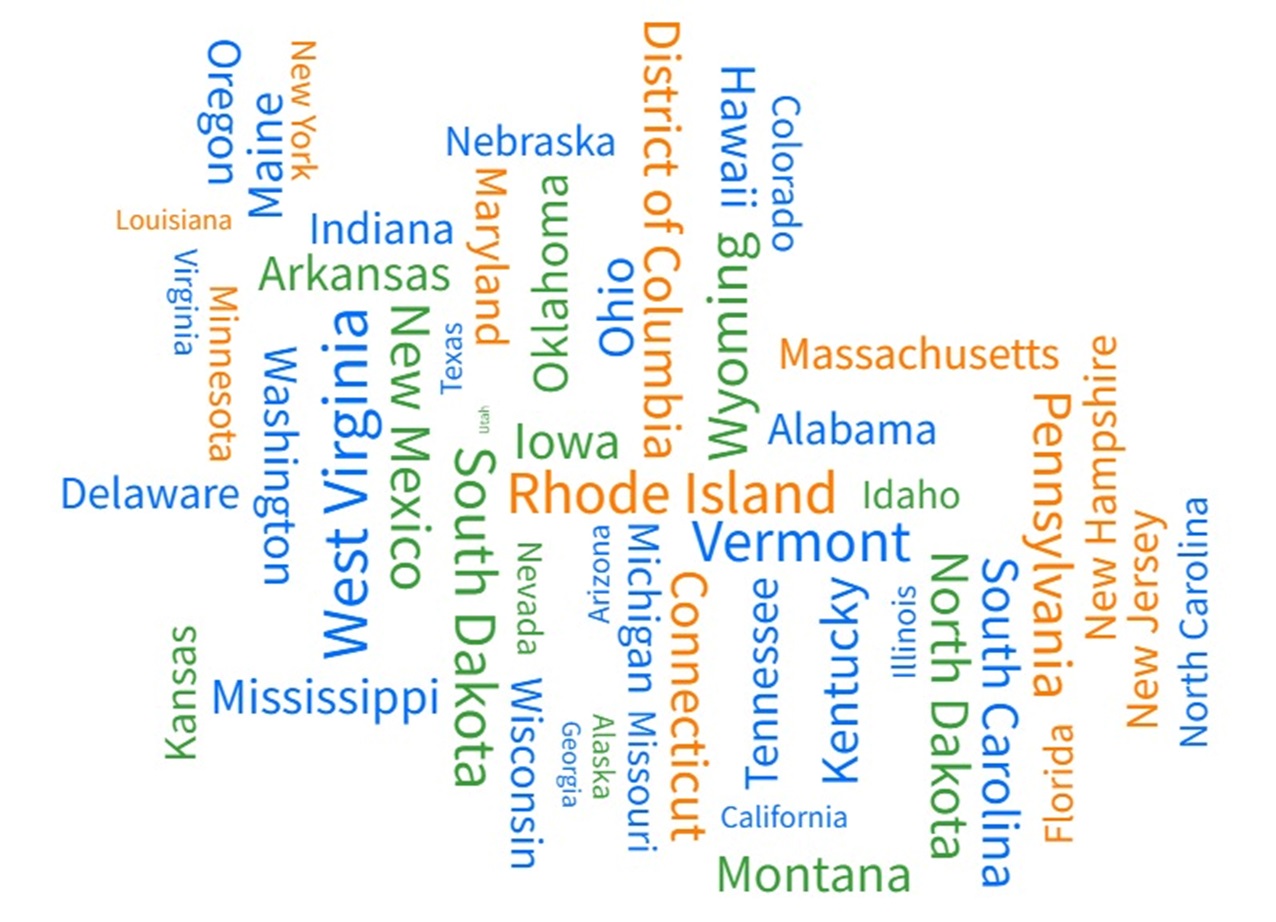
**

**Supplementary Figure 3:** Radial tree map of region-wise stratification of GI physicians’ density quartiles (Purple = West, Blue = South, Pink = Northeast, & Orange = Midwest).

**
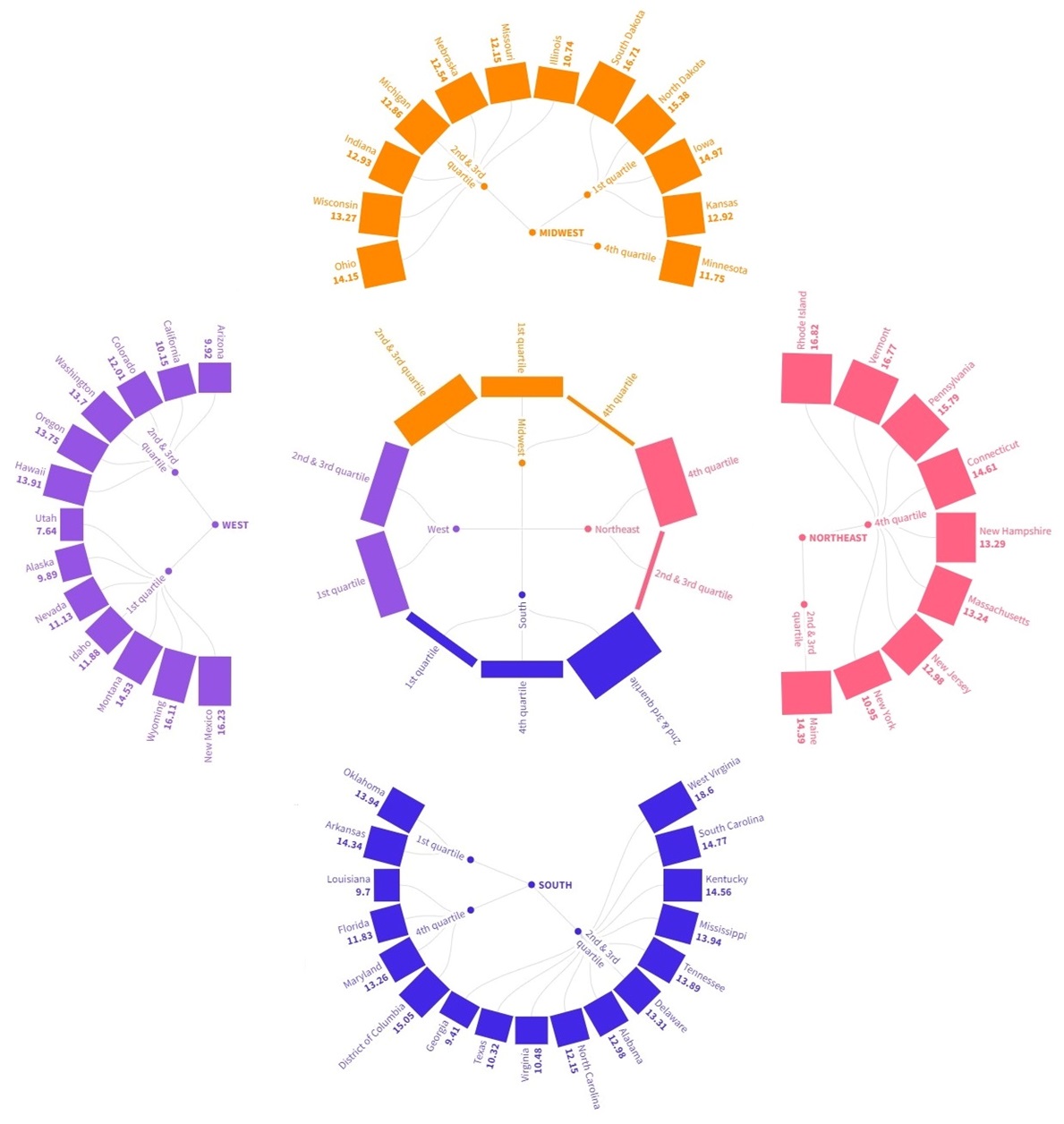
**

**Supplementary Figure 4:** Grouped scatter plot of AHRF GI physician’s density quartiles (coded by colors: 1st quartile – green, 2nd & 3rd quartile – blue, & 4th quartile – orange) with UGIB crude mortality rates among all included counties (CDC WONDER).

**
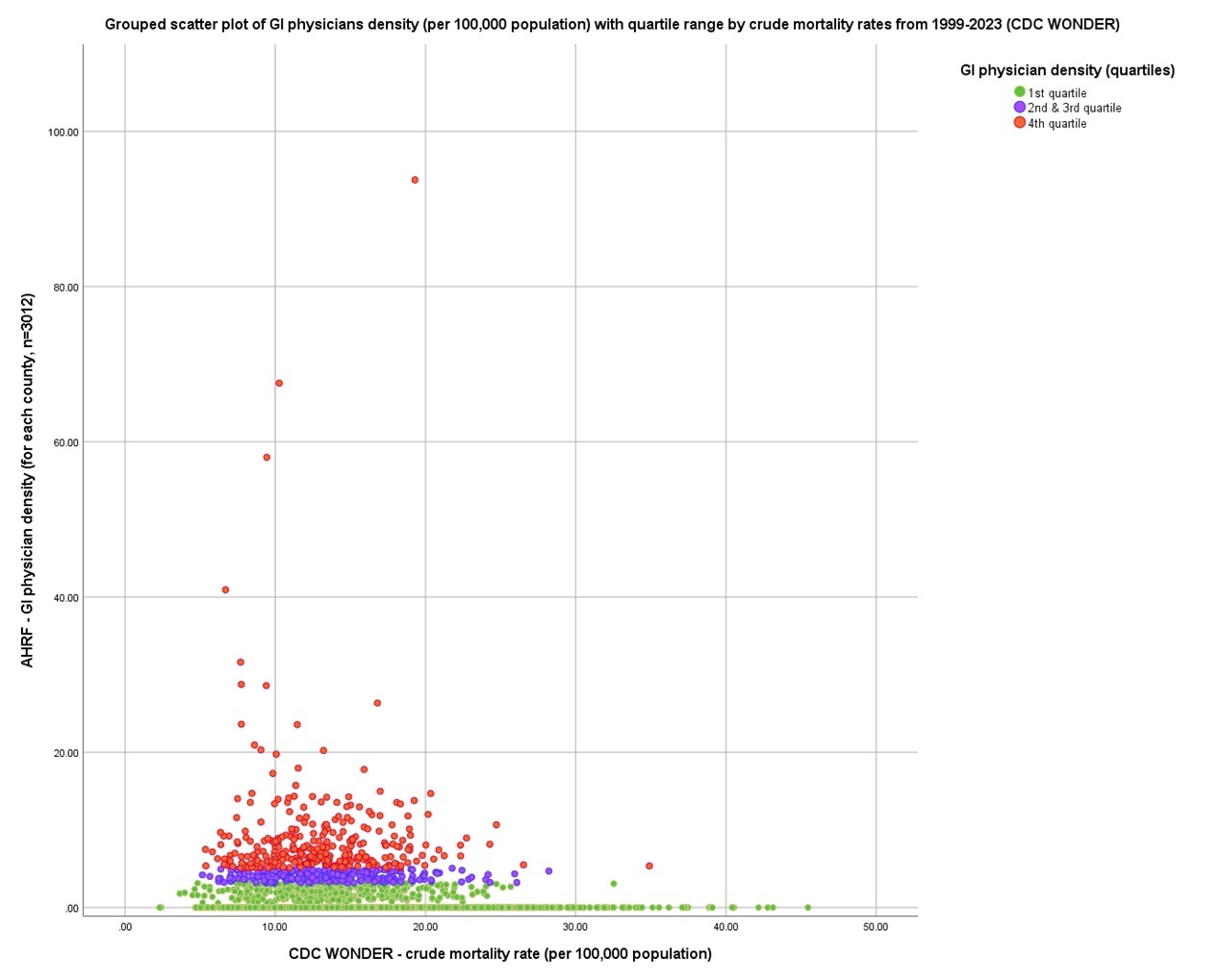
**

1. **Supplemental Tables**

| Supplementary Table 1: ICD-10 codes of upper gastrointestinal bleeding (UGIB).  *** MCD - ICD-10 Codes**:   - I85.0 (Oesophageal varices with bleeding); - K22.6 (Gastro-oesophageal laceration-haemorrhage syndrome); - K25.0 (Gastric ulcer, acute with haemorrhage); - K25.2 (Gastric ulcer, acute with both haemorrhage and perforation); - K25.4 (Gastric ulcer, chronic or unspecified with haemorrhage); - K25.6 (Gastric ulcer, chronic or unspecified with both haemorrhage and perforation); - K26.0 (Duodenal ulcer, acute with haemorrhage); - K26.2 (Duodenal ulcer, acute with both haemorrhage and perforation); - K26.4 (Duodenal ulcer, chronic or unspecified with haemorrhage); - K26.6 (Duodenal ulcer, chronic or unspecified with both haemorrhage and perforation); - K27.0 (Peptic ulcer, site unspecified, acute with haemorrhage); - K27.2 (Peptic ulcer, site unspecified, acute with both haemorrhage and perforation); - K27.4 (Peptic ulcer, site unspecified, chronic or unspecified with haemorrhage); - K27.6 (Peptic ulcer, site unspecified, chronic or unspecified with both haemorrhage and perforation); - K28.0 (Gastrojejunal ulcer, acute with haemorrhage); - K28.2 (Gastrojejunal ulcer, acute with both haemorrhage and perforation); - K28.4 (Gastrojejunal ulcer, chronic or unspecified with haemorrhage); - K28.6 (Gastrojejunal ulcer, chronic or unspecified with both" "haemorrhage and perforation); - K29.0 (Acute haemorrhagic gastritis); - K92.0 (Haematemesis); - K92.1 (Melaena); - K92.2 (Gastrointestinal haemorrhage, unspecified). |
| --- |

| Supplementary Table 2: Counties with missing data and reason for exclusion (n=137). | | | | | | |
| --- | --- | --- | --- | --- | --- | --- |
| County name | State | Not included in AHRF file | Missing in CDC WONDER 1999-2020 files | Missing in CDC WONDER 2021-2023 files | No mortality data available | Estimated population census (according to the AHRF 2020) |
| Wade Hampton Census Area | AK |  | - | - | - | - |
| Kusilvak Census Area | AK | - |  |  | - | 8368 |
| Chugach Census Area | AK | - |  |  | - | 7102 |
| Copper River Census Area | AK | - |  |  | - | 2617 |
| Prince of Wales-Outer Ketchikan Census Area | AK | - | - |  | - | - |
| Skagway-Hoonah-Angoon Census Area | AK | - | - |  | - | - |
| Wrangell-Petersburg Census Area | AK | - | - |  | - | - |
| Bedford city | VA | - | - |  | - | 6222* |
| Clifton Forge city | VA | - | - |  | - | - |
| Aleutians East Borough | AK | - | - | - |  | 3420 |
| Aleutians West Census Area | AK | - | - | - |  | 5232 |
| Bristol Bay Borough | AK | - | - | - |  | 844 |
| Denali Borough | AK | - | - | - |  | 1619 |
| Haines Borough | AK | - | - | - |  | 2080 |
| Hoonah-Angoon Census Area | AK | - | - | - |  | 2365 |
| Lake and Peninsula Borough | AK | - | - | - |  | 1476 |
| Petersburg Borough/Census Area | AK | - | - | - |  | 3398 |
| Skagway Municipality | AK | - | - | - |  | 1240 |
| Wrangell City and Borough | AK | - | - | - |  | 2127 |
| Yakutat Borough | AK | - | - | - |  | 662 |
| Alpine | CA | - | - | - |  | 1204 |
| Custer | CO | - | - | - |  | 4704 |
| Hinsdale | CO | - | - | - |  | 788 |
| Jackson | CO | - | - | - |  | 1379 |
| Kiowa | CO | - | - | - |  | 1446 |
| Mineral | CO | - | - | - |  | 865 |
| San Juan | CO | - | - | - |  | 705 |
| San Miguel | CO | - | - | - |  | 8072 |
| Baker | GA | - | - | - |  | 2876 |
| Chattahoochee | GA | - | - | - |  | 9565 |
| Clay | GA | - | - | - |  | 2848 |
| Echols | GA | - | - | - |  | 3697 |
| Taliaferro | GA | - | - | - |  | 1559 |
| Webster | GA | - | - | - |  | 2348 |
| Kalawao | HI | - | - | - |  | 82 |
| Camas | ID | - | - | - |  | 1077 |
| Clark | ID | - | - | - |  | 790 |
| Teton | ID | - | - | - |  | 11630 |
| Gove | KS | - | - | - |  | 2718 |
| Graham | KS | - | - | - |  | 2415 |
| Hodgeman | KS | - | - | - |  | 1723 |
| Stanton | KS | - | - | - |  | 2084 |
| Wichita | KS | - | - | - |  | 2152 |
| Robertson | KY | - | - | - |  | 2193 |
| Cameron Parish | LA | - | - | - |  | 5617 |
| Nantucket | MA | - | - | - |  | 14255 |
| Red Lake | MN | - | - | - |  | 3935 |
| Issaquena | MS | - | - | - |  | 1338 |
| Worth | MO | - | - | - |  | 1973 |
| Carter | MT | - | - | - |  | 1415 |
| Garfield | MT | - | - | - |  | 1173 |
| Golden Valley | MT | - | - | - |  | 823 |
| Granite | MT | - | - | - |  | 3309 |
| Judith Basin | MT | - | - | - |  | 2023 |
| McCone | MT | - | - | - |  | 1729 |
| Meagher | MT | - | - | - |  | 1927 |
| Petroleum | MT | - | - | - |  | 496 |
| Powder River | MT | - | - | - |  | 1694 |
| Prairie | MT | - | - | - |  | 1088 |
| Sweet Grass | MT | - | - | - |  | 3678 |
| Treasure | MT | - | - | - |  | 762 |
| Wheatland | MT | - | - | - |  | 2069 |
| Arthur | NE | - | - | - |  | 434 |
| Banner | NE | - | - | - |  | 674 |
| Blaine | NE | - | - | - |  | 431 |
| Deuel | NE | - | - | - |  | 1838 |
| Frontier | NE | - | - | - |  | 2519 |
| Gosper | NE | - | - | - |  | 1893 |
| Grant | NE | - | - | - |  | 611 |
| Greeley | NE | - | - | - |  | 2188 |
| Harlan | NE | - | - | - |  | 3073 |
| Hayes | NE | - | - | - |  | 856 |
| Hooker | NE | - | - | - |  | 711 |
| Keya Paha | NE | - | - | - |  | 769 |
| Logan | NE | - | - | - |  | 716 |
| Loup | NE | - | - | - |  | 607 |
| McPherson | NE | - | - | - |  | 399 |
| Rock | NE | - | - | - |  | 1262 |
| Sherman | NE | - | - | - |  | 2959 |
| Sioux | NE | - | - | - |  | 1135 |
| Thomas | NE | - | - | - |  | 669 |
| Wheeler | NE | - | - | - |  | 774 |
| Esmeralda | NV | - | - | - |  | 729 |
| Eureka | NV | - | - | - |  | 1855 |
| Storey | NV | - | - | - |  | 4104 |
| Catron | NM | - | - | - |  | 3579 |
| Harding | NM | - | - | - |  | 657 |
| Billings | ND | - | - | - |  | 945 |
| Emmons | ND | - | - | - |  | 3301 |
| Golden Valley | ND | - | - | - |  | 1736 |
| Oliver | ND | - | - | - |  | 1877 |
| Sheridan | ND | - | - | - |  | 1265 |
| Slope | ND | - | - | - |  | 706 |
| Steele | ND | - | - | - |  | 1798 |
| Harper | OK | - | - | - |  | 3272 |
| Gilliam | OR | - | - | - |  | 1995 |
| Sherman | OR | - | - | - |  | 1870 |
| Wheeler | OR | - | - | - |  | 1451 |
| Buffalo | SD | - | - | - |  | 1948 |
| Campbell | SD | - | - | - |  | 1377 |
| Hanson | SD | - | - | - |  | 3461 |
| Harding | SD | - | - | - |  | 1311 |
| Hyde | SD | - | - | - |  | 1262 |
| Jerauld | SD | - | - | - |  | 1663 |
| Jones | SD | - | - | - |  | 917 |
| McPherson | SD | - | - | - |  | 2411 |
| Sanborn | SD | - | - | - |  | 10280 |
| Stanley | SD | - | - | - |  | 2980 |
| Sully | SD | - | - | - |  | 1446 |
| Ziebach | SD | - | - | - |  | 2413 |
| Borden | TX | - | - | - |  | 631 |
| Collingsworth | TX | - | - | - |  | 2652 |
| Edwards | TX | - | - | - |  | 1422 |
| Foard | TX | - | - | - |  | 1095 |
| Glasscock | TX | - | - | - |  | 1116 |
| Irion | TX | - | - | - |  | 1513 |
| Jeff Davis | TX | - | - | - |  | 1996 |
| Kenedy | TX | - | - | - |  | 350 |
| Kent | TX | - | - | - |  | 753 |
| King | TX | - | - | - |  | 265 |
| Lipscomb | TX | - | - | - |  | 3059 |
| Loving | TX | - | - | - |  | 64 |
| McMullen | TX | - | - | - |  | 600 |
| Menard | TX | - | - | - |  | 1962 |
| Motley | TX | - | - | - |  | 1063 |
| Oldham | TX | - | - | - |  | 1758 |
| Roberts | TX | - | - | - |  | 827 |
| Sherman | TX | - | - | - |  | 2782 |
| Sterling | TX | - | - | - |  | 1372 |
| Stonewall | TX | - | - | - |  | 1245 |
| Terrell | TX | - | - | - |  | 760 |
| Daggett | UT | - | - | - |  | 935 |
| Morgan | UT | - | - | - |  | 12295 |
| Piute | UT | - | - | - |  | 1438 |
| Rich | UT | - | - | - |  | 2510 |
| Manassas Park city | VA | - | - | - |  | 17219 |
| Garfield | WA | - | - | - |  | 2286 |
| * Population census (according to the AHRF 2010). | | | | | | |

| Supplementary Table 3: Trends of crude mortality rates among the gender and age group variables from CDC WONDER database. | | | | | | | | | | | | |
| --- | --- | --- | --- | --- | --- | --- | --- | --- | --- | --- | --- | --- |
| **Year** | **Overall** | **Gender** | | **Age groups (years)** | | | | | | | | |
|  |  | **Female** | **Male** | **<15** | **15-24** | **25-34** | **35-44** | **45-54** | **55-64** | **65-74** | **75-84** | **85+** |
| 1999 | 13.3 | 12.5 | 14.0 | 0.1 | 0.2 | 0.7 | 3.5 | 8.5 | 15.1 | 34.3 | 87.8 | 271.2 |
| 2000 | 13.3 | 12.5 | 14.0 | 0.1 | 0.1 | 0.7 | 3.4 | 8.6 | 15.3 | 32.5 | 88.1 | 273.4 |
| 2001 | 13.0 | 12.3 | 13.6 | 0.1 | 0.1 | 0.6 | 3.3 | 8.5 | 14.4 | 32.4 | 85.2 | 266.3 |
| 2002 | 12.6 | 12.0 | 13.3 | 0.1 | 0.1 | 0.6 | 3.2 | 8.5 | 13.7 | 30.4 | 82.0 | 262.4 |
| 2003 | 12.4 | 11.6 | 13.1 | 0.1 | 0.1 | 0.6 | 3.1 | 8.7 | 13.3 | 30.0 | 78.4 | 251.5 |
| 2004 | 11.7 | 11.1 | 12.4 | 0.1 | 0.2 | 0.5 | 2.8 | 8.2 | 12.9 | 27.6 | 75.6 | 233.5 |
| 2005 | 11.5 | 10.6 | 12.3 | 0.1 | 0.1 | 0.5 | 2.6 | 8.2 | 12.9 | 26.9 | 71.9 | 224.2 |
| 2006 | 11.0 | 10.1 | 11.8 | 0.1 | 0.1 | 0.5 | 2.4 | 7.5 | 12.6 | 24.9 | 67.8 | 213.5 |
| 2007 | 10.6 | 9.6 | 11.4 | 0.1 | 0.1 | 0.5 | 2.3 | 7.5 | 12.5 | 24.0 | 64.3 | 196.6 |
| 2008 | 10.5 | 9.6 | 11.4 | 0.1 | 0.2 | 0.5 | 2.3 | 7.5 | 13.0 | 22.7 | 63.7 | 192.4 |
| 2009 | 10.2 | 9.1 | 11.3 | 0.1 | 0.1 | 0.5 | 2.3 | 7.4 | 13.2 | 22.9 | 59.4 | 177.9 |
| 2010 | 10.5 | 9.4 | 11.6 | 0.1 | 0.1 | 0.6 | 2.3 | 7.5 | 13.6 | 22.8 | 60.0 | 184.1 |
| 2011 | 10.8 | 9.7 | 11.8 | 0.1 | 0.1 | 0.6 | 2.1 | 7.8 | 13.6 | 23.2 | 59.9 | 184.1 |
| 2012 | 10.7 | 9.6 | 11.9 | 0.1 | 0.1 | 0.6 | 2.3 | 7.6 | 14.1 | 22.2 | 59.6 | 176.7 |
| 2013 | 11.1 | 9.9 | 12.3 | 0.1 | 0.1 | 0.5 | 2.4 | 7.6 | 14.6 | 23.0 | 60.3 | 178.6 |
| 2014 | 11.1 | 9.9 | 12.3 | 0.05 | 0.1 | 0.7 | 2.2 | 7.5 | 14.5 | 23.1 | 59.7 | 177.7 |
| 2015 | 11.5 | 10.2 | 12.7 | 0.1 | 0.1 | 0.7 | 2.4 | 7.5 | 14.7 | 24.3 | 59.1 | 179.8 |
| 2016 | 11.8 | 10.4 | 13.3 | 0.1 | 0.1 | 0.8 | 2.4 | 7.5 | 15.7 | 25.3 | 59.0 | 180.0 |
| 2017 | 12.0 | 10.5 | 13.6 | 0.1 | 0.1 | 0.7 | 2.7 | 7.7 | 15.2 | 25.5 | 60.5 | 180.0 |
| 2018 | 12.3 | 10.8 | 13.7 | 0.1 | 0.1 | 0.8 | 2.6 | 7.7 | 15.8 | 25.8 | 59.5 | 178.2 |
| 2019 | 12.4 | 10.7 | 14.0 | 0.1 | 0.1 | 0.9 | 2.9 | 7.4 | 15.9 | 26.2 | 59.7 | 171.2 |
| 2020 | 14.5 | 12.4 | 16.6 | 0.1 | 0.1 | 1.3 | 4.1 | 9.1 | 18.8 | 31.0 | 67.2 | 189.0 |
| 2021 | 15.6 | 13.4 | 17.9 | 0.1 | 0.2 | 1.6 | 4.7 | 10.2 | 20.5 | 33.6 | 75.3 | 209.9 |
| 2022 | 15.0 | 13.0 | 16.9 | 0.1 | 0.2 | 1.4 | 4.3 | 9.2 | 19.0 | 32.9 | 70.1 | 186.4 |
| 2023 | 13.4 | 11.7 | 15.1 | 0.1 | 0.2 | 1.3 | 4.0 | 8.0 | 15.8 | 28.9 | 65.9 | 171.9 |
| **Due to very few mortalities reported, data from <1 years, 1-4 years and 5-14 years from CDC WONDER files were merged together to report as <15 years. Rest all groups were reported 10-years incremented up to 84 years (85+ were also combined).** | | | | | | | | | | | | |

| Supplementary Table 4: Trends of crude mortality rates among the race, census region and urbanization from CDC WONDER database. | | | | | | | | | | | | |
| --- | --- | --- | --- | --- | --- | --- | --- | --- | --- | --- | --- | --- |
| **Year** | **Race** | | | | | **Census Region** | | | | **NCHS Urbanization** | | |
|  | **NH White** | **Black/ African American** | **Asian/ Pacific Islander** | **American Indian/ Alaska Native** | **Hispanic** | **North**  **east** | **Midwest** | **South** | **West** | **Urban** | **Suburban** | **Rural** |
| 1999 | 15.0 | 13.3 | 6.0 | 9.6 | 5.5 | 15.2 | 14.0 | 13.0 | 11.3 | 12.1 | 13.6 | 16.7 |
| 2000 | 15.1 | 13.0 | 5.8 | 10.8 | 5.6 | 15.6 | 13.9 | 12.8 | 11.4 | 12.1 | 13.6 | 16.7 |
| 2001 | 14.9 | 12.6 | 5.8 | 10.9 | 5.4 | 14.9 | 13.8 | 12.5 | 11.3 | 11.8 | 13.6 | 16.1 |
| 2002 | 14.6 | 12.0 | 6.1 | 11.0 | 5.2 | 14.3 | 13.7 | 12.1 | 11.1 | 11.2 | 13.2 | 16.6 |
| 2003 | 14.3 | 11.7 | 5.6 | 11.5 | 5.3 | 13.7 | 13.2 | 12.0 | 11.0 | 10.9 | 13.0 | 16.3 |
| 2004 | 13.7 | 10.9 | 5.3 | 10.6 | 4.9 | 13.2 | 12.3 | 11.2 | 10.7 | 10.4 | 12.2 | 15.5 |
| 2005 | 13.4 | 11.0 | 5.2 | 11.9 | 4.7 | 12.8 | 12.1 | 11.1 | 10.4 | 10 | 12.3 | 15.1 |
| 2006 | 13.0 | 10.1 | 5.0 | 11.3 | 4.4 | 12.3 | 11.7 | 10.5 | 9.9 | 9.5 | 11.9 | 14.4 |
| 2007 | 12.4 | 9.8 | 5.0 | 11.1 | 4.6 | 11.9 | 11.2 | 10.1 | 9.6 | 9.1 | 11.4 | 14.1 |
| 2008 | 12.6 | 9.5 | 4.8 | 12.1 | 4.4 | 11.8 | 11.2 | 10.2 | 9.4 | 9.1 | 11.2 | 14.4 |
| 2009 | 12.2 | 9.5 | 4.7 | 11.2 | 4.5 | 11.4 | 10.8 | 10.0 | 9.2 | 8.8 | 11.1 | 13.8 |
| 2010 | 12.6 | 9.6 | 5.3 | 12.2 | 4.5 | 12.0 | 11.0 | 10.2 | 9.5 | 9.1 | 11.4 | 14.1 |
| 2011 | 13.0 | 9.6 | 5.1 | 12.3 | 4.7 | 12.5 | 11.4 | 10.3 | 9.6 | 9.2 | 11.7 | 14.6 |
| 2012 | 13.0 | 9.5 | 4.7 | 12.7 | 4.8 | 12.1 | 11.2 | 10.5 | 9.6 | 9.2 | 11.8 | 14.4 |
| 2013 | 13.4 | 9.9 | 5.4 | 14.3 | 5.0 | 12.5 | 11.7 | 10.7 | 10.0 | 9.5 | 12.1 | 15.1 |
| 2014 | 13.5 | 9.8 | 5.4 | 15.3 | 5.0 | 12.1 | 11.8 | 11.0 | 10.1 | 9.3 | 12.5 | 15.3 |
| 2015 | 14.0 | 10.1 | 5.4 | 14.6 | 5.2 | 12.5 | 11.9 | 11.2 | 10.6 | 9.6 | 13.0 | 15.5 |
| 2016 | 14.4 | 10.3 | 5.9 | 15.5 | 5.5 | 12.7 | 12.3 | 11.6 | 11.2 | 9.9 | 13.2 | 16.5 |
| 2017 | 14.7 | 10.4 | 6.0 | 15.5 | 5.7 | 12.7 | 12.7 | 11.9 | 11.2 | 10.0 | 13.7 | 16.5 |
| 2018 | 15.1 | 10.7 | 5.8 | 15.6 | 5.7 | 12.8 | 13.1 | 12.3 | 11.1 | 10.1 | 14.0 | 17.2 |
| 2019 | 15.2 | 10.8 | 6.3 | 16.2 | 5.8 | 12.9 | 13.0 | 12.3 | 11.5 | 10.1 | 14.1 | 17.7 |
| 2020 | 17.3 | 13.6 | 7.4 | 24.9 | 7.7 | 14.7 | 15.5 | 14.6 | 13.3 | 12.1 | 16.3 | 20.4 |
| 2021 | 19.0 | 14.8 | 8.3 | 33.2 | 8.5 | 14.9 | 15.7 | 16.1 | 15.2 | 12.9 | 17.6 | 22.5 |
| 2022 | 18.5 | 14.0 | 8.1 | 26.6 | 7.8 | 15.1 | 15.2 | 14.9 | 14.7 | 12.6 | 17.0 | 21.4 |
| 2023 | 16.6 | 12.5 | 7.9 | 21.7 | 6.9 | 13.5 | 13.9 | 13.2 | 13.4 | 11.3 | 15.3 | 19.2 |
| **NH: Non-Hispanic; NCHS: National Center for Health Statistics.** | | | | | | | | | | | | |

| Supplementary Table 5: State-wide age-adjusted mortality rates (AAMR) with CDC crude mortality data (1999-2020 & 2021-2023). | | | | | | | | |
| --- | --- | --- | --- | --- | --- | --- | --- | --- |
| Percentiles | State | AAMR (1999-2020) | Age Adjusted Rate Lower 95% Confidence Interval | Age Adjusted Rate Upper 95% Confidence Interval | State | AAMR (2021-2023) | Age Adjusted Rate Lower 95% Confidence Interval | Age Adjusted Rate Upper 95% Confidence Interval |
| 100 | District of Columbia | 15.6 | 15.0 | 16.3 | Wyoming | 21.3 | 19.3 | 23.3 |
| 98 | New Mexico | 14.6 | 14.2 | 14.9 | South Dakota | 19.3 | 17.7 | 20.9 |
| 96 | West Virginia | 14.6 | 14.3 | 14.9 | New Mexico | 18.3 | 17.3 | 19.3 |
| 94 | Wyoming | 14.1 | 13.4 | 14.8 | Kentucky | 17.4 | 16.8 | 18.1 |
| 92 | Vermont | 13.4 | 12.8 | 14.0 | Colorado | 16.7 | 16.1 | 17.3 |
| 90 | Rhode Island | 13.1 | 12.6 | 13.5 | Montana | 16.0 | 14.8 | 17.2 |
| 88 | South Dakota | 13.0 | 12.5 | 13.5 | Alaska | 15.8 | 14.1 | 17.6 |
| 86 | South Carolina | 12.9 | 12.7 | 13.2 | Mississippi | 15.4 | 14.6 | 16.1 |
| 84 | Alaska | 12.8 | 12.1 | 13.6 | Washington | 15.4 | 14.9 | 15.9 |
| 82 | Mississippi | 12.7 | 12.5 | 13.0 | South Carolina | 15.3 | 14.7 | 15.8 |
| 80 | Kentucky | 12.6 | 12.4 | 12.8 | Oklahoma | 15.2 | 14.6 | 15.9 |
| 78 | North Dakota | 12.4 | 11.9 | 12.9 | Oregon | 14.6 | 14.0 | 15.2 |
| 76 | Tennessee | 12.4 | 12.2 | 12.5 | Vermont | 14.6 | 13.1 | 16.1 |
| 74 | Washington | 12.4 | 12.3 | 12.6 | Tennessee | 14.3 | 13.8 | 14.8 |
| 72 | Oklahoma | 12.3 | 12.1 | 12.6 | Arkansas | 14.2 | 13.5 | 14.9 |
| 70 | Arkansas | 12.1 | 11.8 | 12.3 | Maryland | 14.1 | 13.6 | 14.6 |
| 68 | Maryland | 12.1 | 12.0 | 12.3 | District of Columbia | 13.9 | 12.3 | 15.6 |
| 66 | Ohio | 11.9 | 11.8 | 12.1 | Iowa | 13.9 | 13.2 | 14.5 |
| 64 | Pennsylvania | 11.8 | 11.7 | 12.0 | North Dakota | 13.8 | 12.3 | 15.2 |
| 62 | Colorado | 11.7 | 11.5 | 11.9 | Idaho | 13.5 | 12.6 | 14.5 |
| 60 | Connecticut | 11.6 | 11.4 | 11.8 | Indiana | 13.5 | 13.0 | 14.0 |
| 58 | Alabama | 11.5 | 11.3 | 11.7 | West Virginia | 13.3 | 12.4 | 14.1 |
| 56 | Indiana | 11.5 | 11.3 | 11.7 | Wisconsin | 13.1 | 12.6 | 13.6 |
| 54 | Maine | 11.4 | 11.1 | 11.8 | Kansas | 12.9 | 12.2 | 13.6 |
| 52 | Montana | 11.4 | 11.0 | 11.8 | Rhode Island | 12.8 | 11.7 | 13.9 |
| 50 | Hawaii | 11.2 | 10.9 | 11.6 | Nevada | 12.6 | 12.0 | 13.3 |
| 48 | Iowa | 11.2 | 11.0 | 11.5 | Pennsylvania | 12.6 | 12.2 | 12.9 |
| 46 | North Carolina | 11.2 | 11.1 | 11.4 | Texas | 12.6 | 12.4 | 12.8 |
| 44 | Texas | 11.2 | 11.1 | 11.3 | Delaware | 12.5 | 11.4 | 13.6 |
| 42 | Delaware | 11.1 | 10.7 | 11.6 | Nebraska | 12.3 | 11.4 | 13.1 |
| 40 | Michigan | 11.1 | 11.0 | 11.2 | Ohio | 12.2 | 11.9 | 12.6 |
| 38 | New Hampshire | 11.1 | 10.8 | 11.5 | Minnesota | 12.1 | 11.6 | 12.5 |
| 36 | New Jersey | 11.1 | 11.0 | 11.2 | Alabama | 11.8 | 11.3 | 12.3 |
| 34 | Oregon | 11.1 | 10.9 | 11.4 | New Hampshire | 11.8 | 10.9 | 12.7 |
| 32 | Idaho | 11.0 | 10.7 | 11.4 | Michigan | 11.6 | 11.2 | 11.9 |
| 30 | Kansas | 10.9 | 10.6 | 11.1 | Missouri | 11.5 | 11.1 | 12.0 |
| 28 | Massachusetts | 10.9 | 10.8 | 11.1 | Connecticut | 11.3 | 10.7 | 11.9 |
| 24 | Wisconsin | 10.8 | 10.6 | 10.9 | North Carolina | 11.3 | 11.0 | 11.6 |
| 26 | Nevada | 10.7 | 10.5 | 11.0 | California | 11.0 | 10.8 | 11.2 |
| 22 | Nebraska | 10.5 | 10.2 | 10.8 | Massachusetts | 11.0 | 10.6 | 11.4 |
| 20 | Missouri | 10.2 | 10.0 | 10.4 | New Jersey | 11.0 | 10.7 | 11.4 |
| 18 | Virginia | 10.0 | 9.9 | 10.2 | Utah | 11.0 | 10.3 | 11.7 |
| 16 | California | 9.9 | 9.8 | 9.9 | Georgia | 10.9 | 10.6 | 11.3 |
| 14 | Georgia | 9.9 | 9.8 | 10.1 | Arizona | 10.6 | 10.2 | 11.0 |
| 12 | Minnesota | 9.9 | 9.8 | 10.1 | Louisiana | 10.6 | 10.1 | 11.1 |
| 10 | Illinois | 9.8 | 9.7 | 10.0 | Florida | 10.3 | 10.1 | 10.5 |
| 8 | Utah | 9.7 | 9.4 | 9.9 | Hawaii | 10.2 | 9.4 | 11.0 |
| 6 | New York | 9.3 | 9.2 | 9.4 | Virginia | 10.2 | 9.8 | 10.5 |
| 4 | Louisiana | 9.1 | 8.9 | 9.3 | New York | 9.5 | 9.3 | 9.7 |
| 2 | Arizona | 8.6 | 8.5 | 8.7 | Illinois | 9.3 | 9.0 | 9.6 |
| 0 | Florida | 8.3 | 8.2 | 8.3 | Maine | 7.0 | 6.3 | 7.7 |
| Footnotes: Combined (total) AAMRs for all years could be assessed from CDC WONDER as if they were reported for crude mortality rates (due to limitations in data merging for multiple cause of death files of CDC WONDER datasets for 1999-2023). | | | | | | | | |

| Supplementary Table 6: States in the top 90^th^ percentile of crude UGIB mortality rates overall and stratified according to the urban-rural divide. | | | |
| --- | --- | --- | --- |
| **Overall** | **Large + Fringe Metro** | **Medium + Small Metro** | **Non-metropolitan** |
| West Virginia | Rhode Island | West Virginia | California |
| Rhode Island | Connecticut | Wyoming | West Virginia |
| Vermont | District of Columbia | Pennsylvania | Maryland |
| South Dakota | Pennsylvania | Tennessee | Oregon |
| New Mexico | South Carolina | Delaware | South Dakota |
| Wyoming | Kentucky | Ohio | New Mexico |
| Large central metropolitan + large fringe metropolitan = >1 million population.  Medium metropolitan + Small metropolitan = (250,000-999,999 population and 50,000-249,999 population respectively).  Non-metropolitan: Micropolitan + non-core = (10,000-49,999 population and <10,000 population respectively). | | | |

**References:**

1. Age standardization of death rates: implementation of the year 2000 standard - PubMed [PMID: 9796247]. Available from: https://pubmed.ncbi.nlm.nih.gov/9796247/.

2. Anderson RN, Rosenberg HM. Age standardization of death rates: Implementation of the year 2000 standard. National Vital Statistics Reports; vol 47 no 3. Hyattsville, Maryland. National Center for Health Statistics. 1998.

3. Bishara AJ, Hittner JB. Reducing Bias and Error in the Correlation Coefficient Due to Nonnormality. Educ Psychol Meas. 2015 Oct;75(5):785-804. doi: 10.1177/0013164414557639.
